# Depletion of Retinoic Acid Receptors Initiates a Novel Positive Feedback Mechanism that Promotes Teratogenic Increases in Retinoic Acid

**DOI:** 10.1371/journal.pgen.1003689

**Published:** 2013-08-08

**Authors:** Enrico D'Aniello, Ariel B. Rydeen, Jane L. Anderson, Amrita Mandal, Joshua S. Waxman

**Affiliations:** 1 The Heart Institute, Molecular Cardiovascular Biology and Developmental Biology Divisions, Cincinnati Children's Hospital Medical Center, Cincinnati, Ohio, United States of America; 2 Molecular and Developmental Biology Graduate Program, Cincinnati Children's Hospital Medical Center, Cincinnati, Ohio, United States of America; University of Pennsylvania School of Medicine, United States of America

## Abstract

Normal embryonic development and tissue homeostasis require precise levels of retinoic acid (RA) signaling. Despite the importance of appropriate embryonic RA signaling levels, the mechanisms underlying congenital defects due to perturbations of RA signaling are not completely understood. Here, we report that zebrafish embryos deficient for RA receptor αb1 (RARαb1), a conserved RAR splice variant, have enlarged hearts with increased cardiomyocyte (CM) specification, which are surprisingly the consequence of increased RA signaling. Importantly, depletion of RARαb2 or concurrent depletion of RARαb1 and RARαb2 also results in increased RA signaling, suggesting this effect is a broader consequence of RAR depletion. Concurrent depletion of RARαb1 and Cyp26a1, an enzyme that facilitates degradation of RA, and employment of a novel transgenic RA sensor line support the hypothesis that the increases in RA signaling in RAR deficient embryos are the result of increased embryonic RA coupled with compensatory RAR expression. Our results support an intriguing novel mechanism by which depletion of RARs elicits a previously unrecognized positive feedback loop that can result in developmental defects due to teratogenic increases in embryonic RA.

## Author Summary

Retinoic acid (RA) is the most active metabolic product of Vitamin A. Appropriate levels of RA are required for proper embryonic development and tissue maintenance in all vertebrates. Inappropriate levels of RA in human embryos can cause congenital defects that affect many organs, including the heart and limbs, and lead to numerous types of cancers. Understanding how animals maintain appropriate RA levels and the consequences of inappropriate RA signaling will therefore provide insight into human congenital defects and diseases. RA signaling is mediated by RA receptors (RARs), which are transcription factors that are activated when binding RA. We have found that depletion of RARs in zebrafish results in defects that are surprisingly due to increases in embryonic RA and not a deficiency of RA signaling. Our results are the first to demonstrate that RAR depletion elicits a positive feedback mechanism that promotes RA signaling through complementary increases in both embryonic RA and RAR expression. Therefore, our analysis provides novel insight into the molecular mechanisms that are required to maintain appropriate RA signaling and will positively impact our understanding of the mechanisms underlying congenital defects.

## Introduction

Improper retinoic acid (RA) signaling during development can cause congenital malformations that affect the forelimbs, ocular, cardiovascular, respiratory, urogenital and nervous systems [1]–[4]. Despite almost a century of investigation, the mechanisms underlying many congenital defects due to fluctuations in RA signaling are still not understood. RA acts as a ligand for RA receptors (RARs), members of the nuclear hormone family of transcription factors [5]. Work using disparate embryonic models has provided critical insight into the molecular mechanisms and developmental requirements of RAR function in vertebrate embryos [6]–[12]. In addition, RAR deficiency and inappropriate RA signaling are associated with numerous types of cancers [13]. In the majority of cases, the mechanism by which loss of RARs promote tumorigenesis is not understood. Therefore, understanding the roles of RARs during development will help elucidate the mechanisms underlying congenital defects, and possibly cancers, caused by inappropriate RA signaling [3], [4].

RA signaling employs a number of feedback mechanisms in order to maintain appropriate levels in the embryo and tissues. The best characterized feedback mechanism is through regulation of the RA producing [retinol dehydrogenases (RDHs) and retinaldehyde dehydrogenases (Aldh1a)] and degrading (Cyp26) enzymes. Specifically, increased RA signaling inhibits the expression of the RA producing enzymes, while promoting Cyp26a1 expression. Conversely, decreased RA signaling promotes expression of the RA producing enzymes, while inhibiting Cyp26a1 expression [14]–[18]. While modulation of RA signaling also affects the expression of other factors that control RA signaling [5], [19], less well understood are feedback mechanisms that may influence RAR expression. RA response elements (RAREs) have been found in murine RARα2 and RARβ2 promoters and RARβ2 has been shown to be RA responsive 20–22. However, if decreases in RA signaling, in particular due to loss of RAR expression, lead to compensatory expression of other RARs is less clear. While initial studies of mouse RAR KO mice suggested that there was not compensatory RAR expression in RAR deficient mice [11], [12], more recent studies using siRNA to deplete RARα have challenged this model and suggested that there may be compensatory RAR expression in RARα deficient embryos [23]. Therefore, if there are RA feedback mechanisms that influence RAR expression and how the employment of these feedback mechanisms impact embryonic development are not well understood.

Here, we find that depletion of RARαb1, a previously unrecognized yet conserved zebrafish RARα splice variant, causes an increase in CM specification and heart size, which is due to the triggering of a feedback mechanism that surprisingly promotes increased RA signaling from surplus embryonic RA and compensatory RAR expression. Our results provide insight into a newly recognized positive feedback mechanism that we posit resists fluctuations in RA signaling due to perturbation in RAR expression. However, if improperly maintained, the positive feedback can result in RA induced congenital defects. Altogether, the results from this study significantly enhance our understanding of the feedback mechanisms that are used to maintain appropriate RA signaling levels and previously unexplored mechanisms that potentially underlie congenital defects.

## Results

### RARαb1 deficient embryos have enlarged hearts and increased CM specification

**Figure 1:**
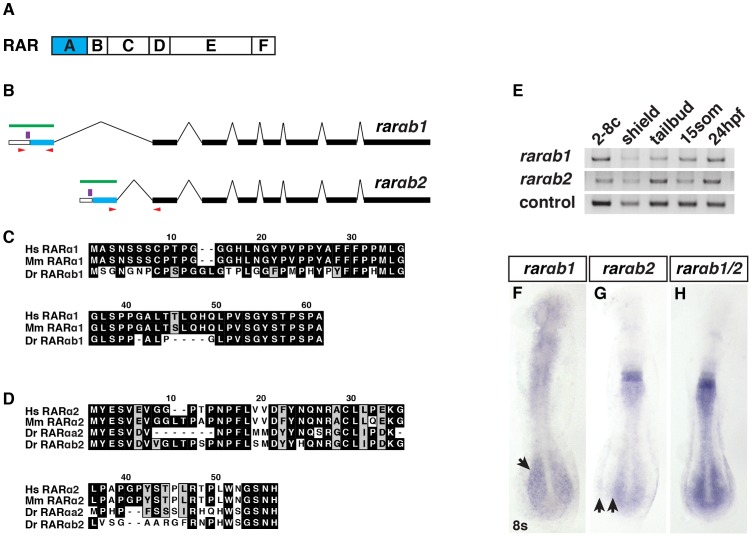
RARαb1 and RARαb2 sequences and expression. (A) Schematic representation of RAR domains. Blue box indicates the variable A domain, which is different between RARαb1 and the previously identified RARαb2 splice variant. (B) Schematic representation of RARαb1 and RARαb2 genomic organization (adapted from Ensemble_v9). Blue bars represent the first exon, which encodes the respective A domains. White bars represent the 5′ UTRs. Black bars represent the exons that are common to the two variants. Green bars represent the target of the antisense probes used for ISH. Red arrows indicate the position of the primers used to perform RT-PCR. Purple bars indicate the position of the morpholino target sequences. (C) Alignments of the A domains of human (Hs) RARα1, mouse (Mm) RARα1, and zebrafish (Dr) RARαb1. The presence of this previously unrecognized splice variant was recently confirmed in the latest zebrafish genome assembly (Ensemble Zv9). There is no RARαa splice variant 1 ortholog in the zebrafish genome. (D) Alignments of the A domains of Hs RARα2, Mm RARα2, Dr RARαa2, and Dr RARαb2. (E) Reverse transcriptase PCR (RT-PCR) for the zebrafish *rarαb* isoforms. *max* was used as the control. -RT control did not reveal genomic contamination (data not shown). (F) *Rarαb*1 is expressed in the ventral anterior of the embryo and the presomitic paraxial mesoderm (arrow) at the 8 somite (s) stage. (G) *Rarαb*2 is expressed in rhombomeres 5 and 6, the spinal cord and the posterior lateral plate mesoderm (LPM). Arrows indicate the space between the posterior spinal cord and LPM expression domains. (H) Together, the expression patterns recapitulate a previously reported *rarαb* probe (referred to as *rarαb1/2*), which detects both isoforms [24]. In F–H, embryos are flatmounted and are dorsal views with anterior up.

In contrast to the studies of RARs in mice [9]–[12], depletion of RARs has not been able to recapitulate all of the consequences of loss of RA signaling in zebrafish [8], which prompted us to determine if additional conserved RAR variants exist in zebrafish beyond what has already been reported [24]. We cloned a previously unrecognized RARα splice variant that is orthologous to human, mouse and *Xenopus* RARα1 termed RARαb1 (Figure 1A–1C). The previously cloned zebrafish RARα homologs RARαa and RARαb are teleost specific paralogs and both are orthologous to the splice variant 2 found in tetrapods (Figure 1B, 1D) [24]. Both *rarαb1* and *rarαb2* are expressed maternally and zygotically (Figure 1E), with ubiquitous expression until the tailbud stage (Figure S1A–S1I). After the tailbud stage, their expression patterns deviate (Figure 1F–1H and Figure S1J–S1O).

**Figure 2:**
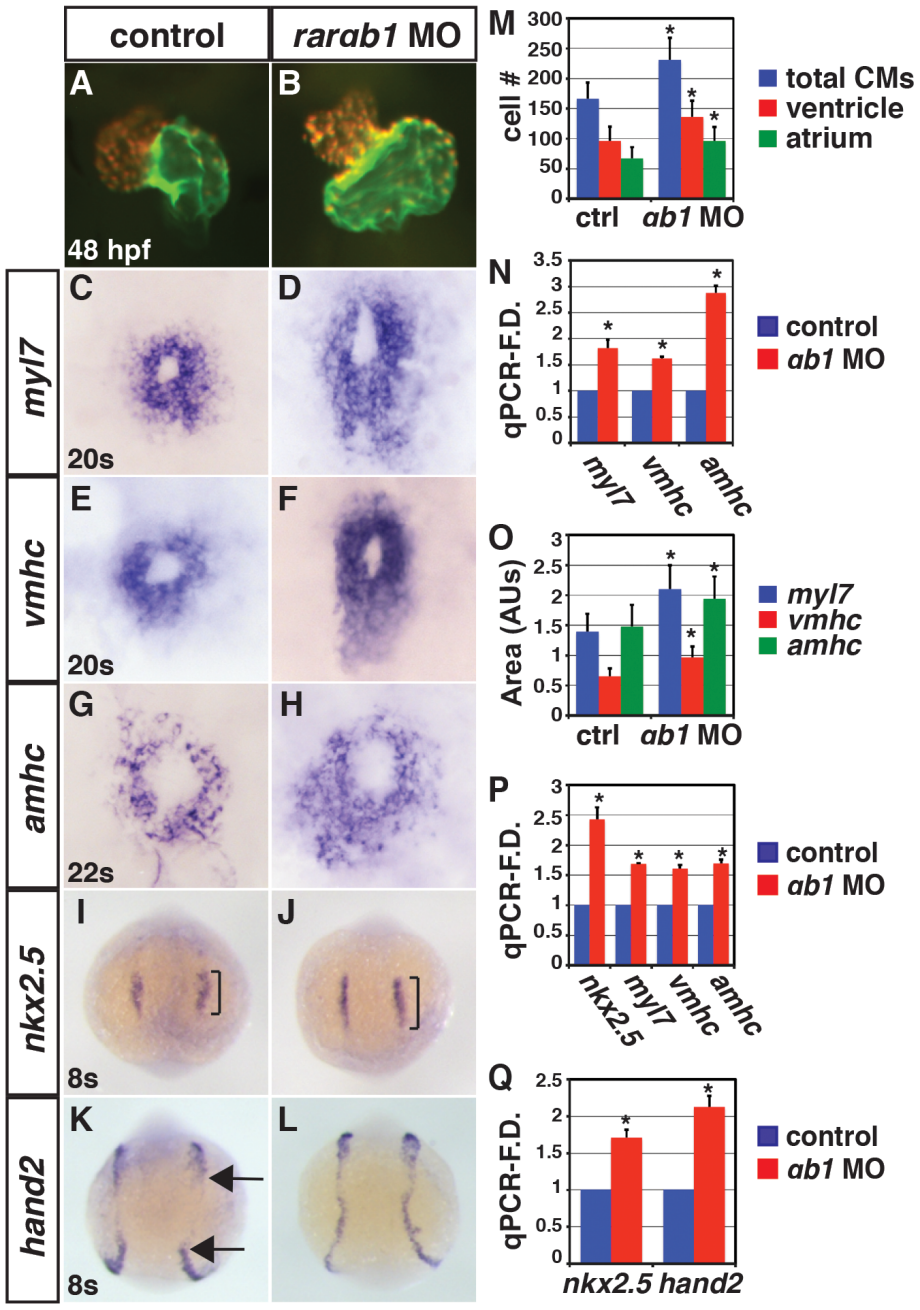
RARαb1 deficient embryos have enlarged hearts with increased CM number. (A, B) Hearts from control sibling and RARαb1 deficient *Tg(-5.1myl7:DsRed-NLS)^f2^* embryos. Images are frontal views. Red indicates ventricle. Green indicates atrium. (C–H) ISH for CM differentiation marker genes. (I–L) ISH for CM progenitor marker genes. Brackets in I and J indicate length of *nkx2.5* expression. Arrows in K indicate posterior and anterior limits of the *hand2* expression domains in the LPM. In C–L, views are dorsal with anterior up. (M) Mean CM number at 48 hpf. (N) qPCR for CM differentiation marker gene expression at 48 hpf. (O) Areas of the amount of cells expressing the CM differentiation marker genes at the 20 s and 22 s stages. (P) qPCR for CM differentiation marker gene and *nkx2.5* expression at 24 hpf. (Q) qPCR for CM progenitor gene expression at the 8 s stage. Asterisk in all graphs indicate a statistically significant difference compared to controls (p<0.05). Error bars in all graphs indicate standard deviation.

We used a translation blocking morpholino (MO) to examine the function of RARαb1 (Figure 1B). By 48 hours post-fertilization (hpf), RARαb1 deficient embryos had enlarged hearts with increased CM number and expression of CM marker genes *myl7*, *vmhc* and *amhc* (Figure 2A, 2B, 2M, 2N and Figure S2A–S2D). Similar increases in CM number were also found at 55 hpf (Figure S3A–S3C), suggesting the major addition of surplus CMs occurs during earlier stages of development. Consistent with this idea, we observed an expansion of CM differentiation (*myl7*, *vmhc*, and *amhc*) and progenitor (*nkx2.5* and *hand2*) marker expression in RARαb1 deficient embryos at earlier stages via in situ hybridization (ISH) and quantitative real-time PCR (qPCR; Figure 2C–2L, 2O–2Q). Injecting the RARαb1 MO along with *rarαb1* mRNA that lacks the 5′UTR MO binding sequence is able to rescue the increased heart size, supporting the specificity of the phenotype (Figure S4A–S4D). Together, these results suggest that RARαb1 deficient embryos have increased CM specification, number and heart size.

### Depletion of zebrafish RARαb paralogs promotes RA signaling

**Figure 3:**
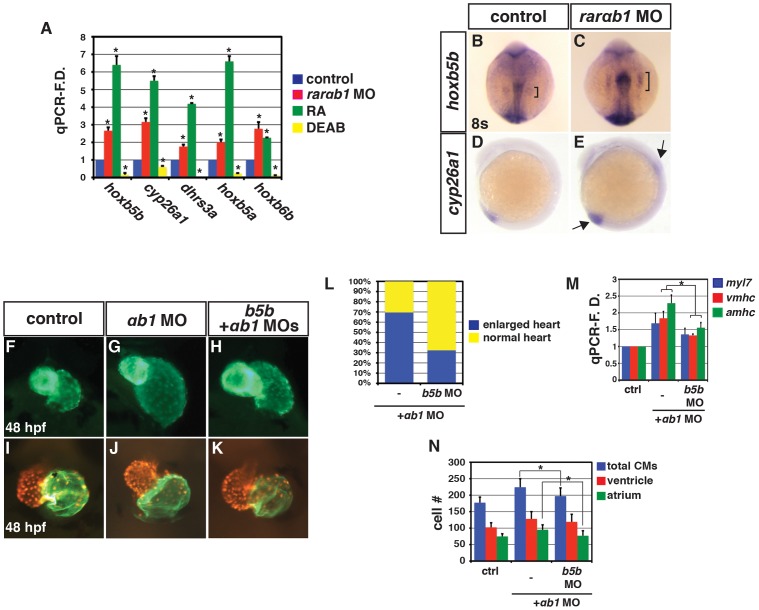
RARαb1 deficient embryos have increased expression of RA signaling responsive genes. (A) qPCR for RA signaling responsive gene expression at the 8 s stage. (B, C) ISH for *hoxb5b* expression at the 8 s stage. Bracket indicates length of expression in the LPM. Views are dorsal with anterior up. (D, E) ISH for *cyp26a1* expression at the 8 s stage. Arrows in E indicate increased expression in the tailbud and spinal cord. Views are lateral with anterior up and dorsal right. (F–H) Fronto-lateral views of *Tg(-5.1myl7:GFP)^f2^* embryos at 48 hpf of control sibling, RARαb1 deficient embryos, and RARαb1+Hoxb5b deficient embryos. (I–K) Hearts from control sibling, RARαb1 deficient embryos and RARαb1+Hoxb5b deficient *Tg(-5.1myl7:DsRed-NLS)^f2^* embryos. Images are frontal views. Red indicates ventricle. Green indicates atrium. (L) Percentage of *control*+*rarαb1* MOs (n = 60), *hoxb5b*+*rarαb1* MOs (n = 68) showing enlarged and normal hearts. (M) qPCR for CM differentiation gene expression at 48 hpf. (N) Mean CM number at 48 hpf.

The increased atrial and ventricular CM number in RARαb1 deficient embryos are reminiscent of RA signaling deficient embryos [25], [26]. Therefore, we examined *hoxb5b* expression, which functions downstream of RA signaling to restrict atrial CM number [26] and is likely a direct target of RARs (Figure S5A–S5D). Unexpectedly, we found that *hoxb5b* expression was increased in RARαb1 deficient embryos (Figure 3A–3C). While this was initially perplexing, our recent studies showed that Hoxb5b overexpression is able to mimic many of the teratogenic effects of RA treatment [27]. Therefore, we asked if the increases in *hoxb5b* expression in RARαb1 deficient embryos could be a cause of the enlarged hearts. While depletion of *hoxb5b* alone using a low concentration of *hoxb5b* MO does not affect CM number (Figure S6A–S6C), we found that concurrent depletion of RARαb1 and Hoxb5b largely restored heart morphology, CM differentiation marker expression, and CM number relative to the RARαb1 deficient embryos (Figure 3F–3N), suggesting that the increased CM number in RARαb1 deficient embryos is in part a consequence of the increased *hoxb5b* expression.

We next examined the expression of additional RA signaling responsive genes. Similar to *hoxb5b*, we found that the expression of additional RA signaling responsive genes, including *cyp26a1*, *dhrs3a*, *hoxb6b* and *hoxb5a*, was increased in RARαb1 deficient embryos (Figure 3A). Comparing RA responsive gene expression in RA treated and RARαb1 deficient embryos, we found that the trends were similar, but that RA treatment typically induced a greater increase in expression (Figure 3A). Conversely, treatment with DEAB, an antagonist of the RA producing enzyme Aldh1a, inhibited RA responsive gene expression (Figure 3A). These findings indicate that RARαb1 depletion paradoxically results in increased expression of RA signaling responsive genes.

**Figure 4:**
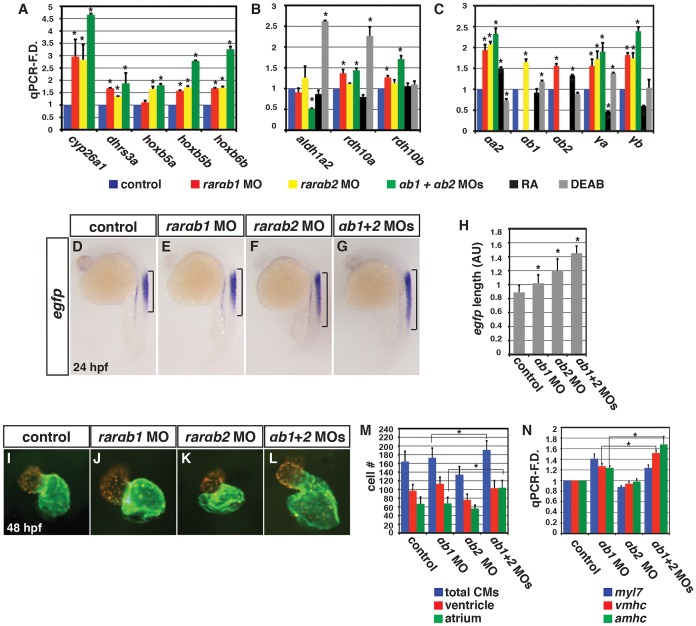
Concurrent depletion of RARαb1 and RARαb2 promotes increased RA signaling and atrial CM number. qPCR for (A) RA signaling responsive gene, (B) RA metabolizing gene, and (C) zebrafish *rar* expression in control sibling, RARαb1 deficient, RARαb2 deficient, RARαb1+RARαb2 (suboptimal doses) deficient, RA treated, and DEAB treated embryos at the 8 s stage. (D–G) ISH for *egfp* expression in *Tg(12XRARE-ef1a:EGFP)^sk72^* embryos. Brackets indicate the length of *egfp* expression in the spinal cord. (H) Measurements of the length in arbitrary units (AU) of *egfp* expression in the spinal cord of *Tg(12XRARE-ef1a:EGFP)^sk72^* embryos. (I–L) Hearts from control and RARαb depleted *Tg(-5.1myl7:DsRed-NLS)^f2^* embryos. Images are frontal views. Red indicates ventricle. Green indicates atrium. (M) Mean CM number from *Tg(-5.1myl7:DsRed-NLS)^f2^* hearts at 48 hpf. (N) qPCR for CM marker gene expression at 48 hpf. While modest increases in *vmhc* expression in RARαb1+RARαb2 deficient embryos were observed relative to RARαb1 (suboptimal dose) deficient embryos, corresponding increases in ventricular CM number were not observed.

We next wanted to determine if increases in RA signaling responsive genes were specific to RARαb1 depletion, so we examined RA responsive gene expression in RARαb2 deficient embryos. Previous studies found that RARαb2 deficient embryos lack forelimbs (pectoral fins) and *tbx5a* expression [8], [28], which we confirmed (Figure S7A, S7C, S7D, S7F, S7H, S7I). However, similar to RARαb1 depletion (Figure 3A and Figure 4A), RARαb2 deficient embryos had increased expression of RA signaling responsive genes (Figure 4A). While the previous studies found a loss of forelimbs, defects in heart development were not reported. Despite the loss of forelimbs and increase in RA signaling responsive genes, we did not observe an increase in heart size, CM number or CM gene expression (Figure S8A–S8D). Therefore, although eliciting similar increases in RA signaling responsive gene expression, individual depletion of RARαb1 and RARαb2 results in distinct defects.

To determine the functional consequences of concurrent RARαb1 and RARαb2 depletion, we co-injected a suboptimal dose of each MO. Unfortunately, co-injection of an optimal dose of each MO resulted in significant non-specific toxicity even when injected along with *p53* MO. However, concurrent depletion of the RARαbs using suboptimal MO doses resulted in a dramatic increase in RA signaling responsive genes, above what was seen with depletion of RARαb1 and RARαb2 alone using the optimal MO doses (Figure 4A). Additionally, there was an anterior shift of *hoxb5a* expression in the spinal cord of RARαb1+2 deficient embryos, suggesting the spinal cords are posteriorized (Figure S9A–S9E). Increased RA signaling inhibits *aldh1a2* expression through a negative feedback mechanism 16–18. Although *aldh1a2* expression in individual RARαb1 and RARαb2 deficient embryos was not suppressed (Figure 4B), *aldh1a2* expression was decreased in embryos depleted for both RARαb variants (Figure 4B). To corroborate the increases in endogenous RA signaling responsive genes, we used the RA signaling reporter line *Tg(12XRARE-ef1a:EGFP)^sk72^* 29. Again, co-depletion of both RARαbs resulted in a greater expansion of *egfp* expression, compared to the individual depletion of each RARαb (Figure 4D–4H). Therefore, these experiments support the hypothesis that the RARαb1+2 deficient embryos are sensing more significant increases in RA signaling than embryos deficient for either RARαb variant alone.

We next examined the consequences of this functional interaction on heart development. We found that the hearts of RARαb1+2 deficient embryos had increased atrial size, CM number, and a dramatic increase in *amhc* expression (Figure 4I, 4L–4N and Figure S10A–S10D). Significant effects on CM number or heart size were not found when using a suboptimal dose of either RARαb1 or RARαb2 MO alone (Figure 4I–4K, 4M), though we did find a modest increase in CM marker gene expression in the RARαb1 deficient embryos (Figure 4N). Interestingly, in RARαb1+2 deficient embryos we found more significant increases in atrial CM number and *amhc* expression (Figure 4M, 4N), which were remarkably similar to the consequences of modest increases in RA signaling due to RA treatment 27. Increased RA signaling can also inhibit forelimb development 17 and RARαb1 deficient embryos also have smaller forelimbs and a modest reduction of *tbx5a* expression (Figure S7A, S7B, S7D, S7F, S7G, S7I). A functional interaction with the RARαb variants that resulted in loss of forelimbs was also observed (Figure S7D, S7E). Therefore, concurrent depletion of RARαb variants elicits increases in RA signaling with heart and forelimb phenotypes that are strikingly similar to increases in RA signaling caused from RA treatment.

### Rarαb1 Deficient Embryos Have Increased Embryonic Ra

We sought to understand the mechanism underlying the increase in RA signaling in RARαb deficient embryos. In the absence of RA, RARs are thought to interact with transcriptional co-repressors, while binding of RA converts the RARs to transcriptional activators 1,5. A previous study in *Xenopus* suggested that RARs are required as transcriptional repressors in some developmental contexts 6. However, our gain-of-function analysis did not support that these zebrafish RARs function as transcriptional repressors (Figure S11A–S11L), consistent with what we have reported previously 29. However, Manshouri et al. 23 found a compensatory increase in the expression of other RARs when using siRNA to deplete RARα in mice. Similarly, we found that the expression of other zebrafish RARs 24 was increased in RARαb deficient embryos (Figure 4C and Figure S12A–S12L), suggesting that compensatory RAR expression is a conserved response to depletion of RARα homologs in vertebrates. Although Manshouri et al. 23 proposed the compensatory RAR expression was RA signaling dependent, our results suggest that the expression of most RARs is potentially regulated independent of RA signaling (Figure 4C), because the effects on RAR expression did not parallel modulation of RA signaling using RA and DEAB. While we observed compensatory expression of other RARs in RARαb deficient embryos, it is difficult to conclude that increased RAR expression is the sole cause of the increase in RA signaling since overexpression of RARs in zebrafish embryos does not produce significant positive or negative effects on RA responsive gene expression (Figure S11A–S11J) 29. Nevertheless, our results suggest that when depleting RARαbs in zebrafish embryos compensatory RARs are present that can mediate RA signaling.

Because we did not have evidence that RARs act as transcriptional repressors or that the increased expression of RARs alone contributes to the increases in RA signaling in RARαb deficient embryos, we hypothesized that the depletion of RARs may trigger an increase in embryonic RA. Although *aldh1a2* expression was suppressed in RARαb1+2 deficient embryos similar to when embryos sense increases in RA signaling (Figure 4B) 16–18, the expression of *rdh10a* and *rdh10b*, which control a limiting step in RA production in vertebrates by generating retinal from retinol 14,15, was increased in RARαb1 and RARαb1+2 depleted embryos (Figure 4B and Fig. S13A–S13C). Interestingly, *rdh10b* expression, which was not sensitive to modulation of RA signaling, was increased in RARαb deficient embryos (Figure 4B). Therefore, our results suggest that depletion of RARαbs triggers an increase in RA through promoting *rdh10* expression.

**Figure 5:**
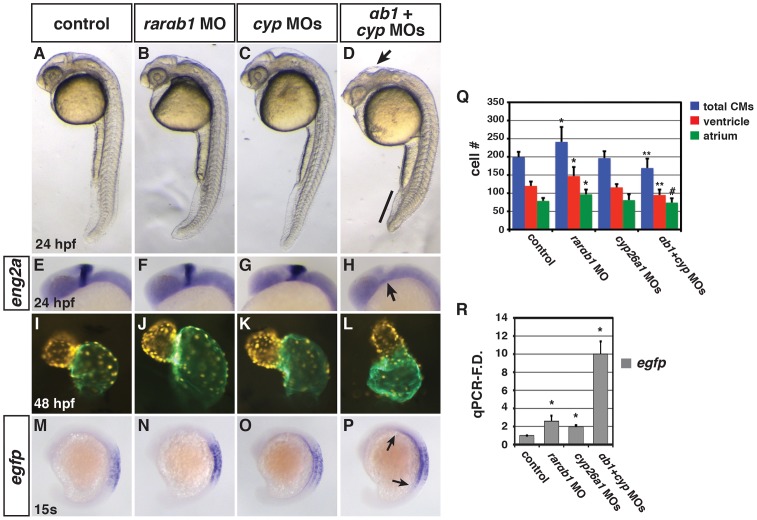
Concurrent depletion of RARαb1 and Cyp26a1 results in phenotypes resembling RA treatment. (A–D) Control sibling, RARαb1 deficient, Cyp26a1 deficient, and RARαb1+Cyp26a1 deficient embryos. A suboptimal dose of the *cyp26a1* MOs was used that did not cause ostensible defects for these experiments. In D, arrow indicates loss of the MHB and line indicates shortened tail. Images are lateral views with dorsal right and anterior up. (E–H) ISH for *eng2a*, which marks the MHB. 100% of (E) control sibling (n = 11), (F) RARαb1 deficient (n = 7), and (G) Cyp26a1 deficient (n = 7) had *eng2a* expression. 85% of (H) RARαb1+Cyp26a1 deficient embryos (n = 7) had a complete absence of *eng2a* expression (arrow in H). Equivalent results were obtained using *pax2a*, which also marks the MHB (data not shown). (I–L) Hearts from control sibling, RARαb1 deficient, Cyp26a1 deficient, and RARαb1+Cyp26a1 deficient *Tg(-5.1myl7:DsRed-NLS)^f2^* embryos. Images are frontal views. Red indicates ventricle. Green indicates atrium. (M–P) ISH for *egfp* in *Tg(β-actin:GDBD-RLBD)^cch1^*;*Tg(UAS:EGFP)* embryos. Lateral views with dorsal right and anterior up. (Q) Mean CM number at 48 hpf and (R) qPCR for *egfp* expression at 15 s in control sibling, RARαb1 deficient, Cyp26a1 deficient, and RARαb1+Cyp26a1 deficient embryos. Double asterisks in Q indicate a statistically significant difference relative to control and RARαb1 deficient embryos. Pound sign in Q indicates a statistically significant difference relative to RARαb1 deficient embryos.

In addition to inhibiting *aldh1a2* expression, increased RA signaling promotes a negative feedback mechanism that limits RA levels by positively regulating Cyp26a1 expression 16–18. Since we observe an increase in *cyp26a1* expression in RARαb1 deficient embryos (Figure 3A, 3D, 3E and Figure 4A), which was also consistent with the hypothesis that there is increased embryonic RA, we postulated that the increased Cyp26a1 may be protecting the RARαb1 deficient embryos from teratogenic increases in embryonic RA. Therefore, we concurrently depleted RARαb1 and Cyp26a1 to determine if there was a functional interaction indicative of increased embryonic RA. For these experiments, a suboptimal dose of *cyp26a1* MOs (Figure S14A–S14E) was used to more easily discern a functional interaction. In either the RARαb1 or Cyp26a1 deficient embryos alone, we never observed absence of the MHB or defects in tail elongation (Figure 5A–5C, 5E–5G). However, co-depletion of RARαb1 and Cyp26a1 resulted in a loss of the MHB and truncated tails (Figure 5D, 5H), similar to increases in RA signaling 17,19,29,30. Furthermore, we found that RARαb1+Cyp26a1 deficient embryos had dismorphic hearts with a specific reduction in ventricular CM number compared to controls embryos hearts (Figure 5I–5L, 5Q), which interestingly resembles the trend we previously found in embryos with intermediate increases in RA signaling 27.

**Figure 6:**
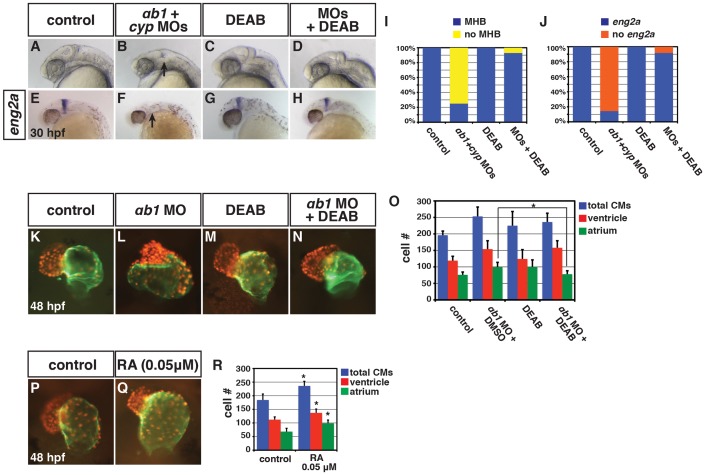
Reduction of RA in RARαb1 deficient embryos can rescue developmental defects. (A–H) Control sibling, RARαb1+Cyp26a1 deficient, control sibling treated with DEAB, and RARαb1+Cyp26a1 treated with DEAB embryos. In B and F, arrows indicates loss of the MHB and *eng2a* expression. Images are lateral views with dorsal right and anterior up. (I) Percentage of control sibling (n = 16), RARαb1+Cyp26a1 deficient embryos (n = 16), control sibling embryos treated with DEAB (n = 13), and RARαb1+Cyp26a1 deficient embryos treated with DEAB (n = 14) that had a MHB based on morphology. (J) Percentage of control sibling (n = 17), RARαb1+Cyp26a1 deficient embryos (n = 14), control sibling embryos treated with DEAB (n = 15), and RARαb1+Cyp26a1 deficient embryos treated with DEAB (n = 12) that had *eng2a* expression at the MHB. (K–N) Hearts from *Tg(-5.1myl7:DsRed-NLS)^f2^* control sibling, RARαb1 deficient, DEAB treated, and DEAB+RARαb1deficient embryos. Images are frontal views. Red indicates ventricle. Green indicates atrium. (O) Mean CM number at 48 hpf. (P,Q) Hearts from *Tg(-5.1myl7:DsRed-NLS)^f2^* control sibling embryos and *Tg(-5.1myl7:DsRed-NLS)^f2^* embryos treated with a low concentration of RA. Images are frontal views. Red indicates ventricle. Green indicates atrium. (R) Mean CM number at 48 hpf.

Although one interpretation of the functional interaction of RARαb1 and Cyp26a1 depletion is that there is increased embryonic RA levels in these embryos, we wanted to further test this hypothesis using additional assays. First, we sought to use a distinct readout of embryonic RA, so we made a novel stable transgenic RA sensor line which incorporated the RARαb ligand binding domain (RLBD) fused to the Gal4 DNA binding domain (GDBD) expressed under the β-actin promoter (Figure S15A–S15G) 31. Previous studies have found that similar GDBD fusions with nuclear hormone receptor LBDs create an effective reporter of nuclear hormone activity 6,32,33. We observed a dramatic increase in reporter expression when RARαb1 and Cyp26a1 were depleted together in *Tg(β-actin:GDBD-RLBD)*; *Tg(UAS:EGFP)* embryos (Figure 5M–5P, 5R) 34. Second, our hypothesis predicted that reducing embryonic RA levels should be able to rescue teratogenic phenotypes found in RARαb1+Cyp26a1 and RARαb1 deficient embryos. Consistent with this hypothesis, DEAB treatment of RARαb1+Cyp26a1 deficient embryos was able to rescue the loss of MHB (Figure 6A–6J). Additionally, treatment of RARαb1 deficient embryos with DEAB partially rescue the enlarged heart phenotype and restored atrial CM number (Figure 6K–6O). Lastly, our hypothesis predicts that exogenous treatment with a concentration of RA that causes a minor increase in RA signaling should result in aberrant heart phenotypes that are similar to RARαb1 deficient embryos. Indeed, embryos treated with low concentrations of exogenous RA (lower than we had reported using previously 27) had enlarged hearts with an increase in both atrial and ventricular CM number at 48 hpf (Figure 6P–6R). Altogether, our results suggest that increases in embryonic RA, coupled with compensatory RAR expression, contribute to the developmental defects found in RARαb1 deficient embryos.

## Discussion

**Figure 7:**
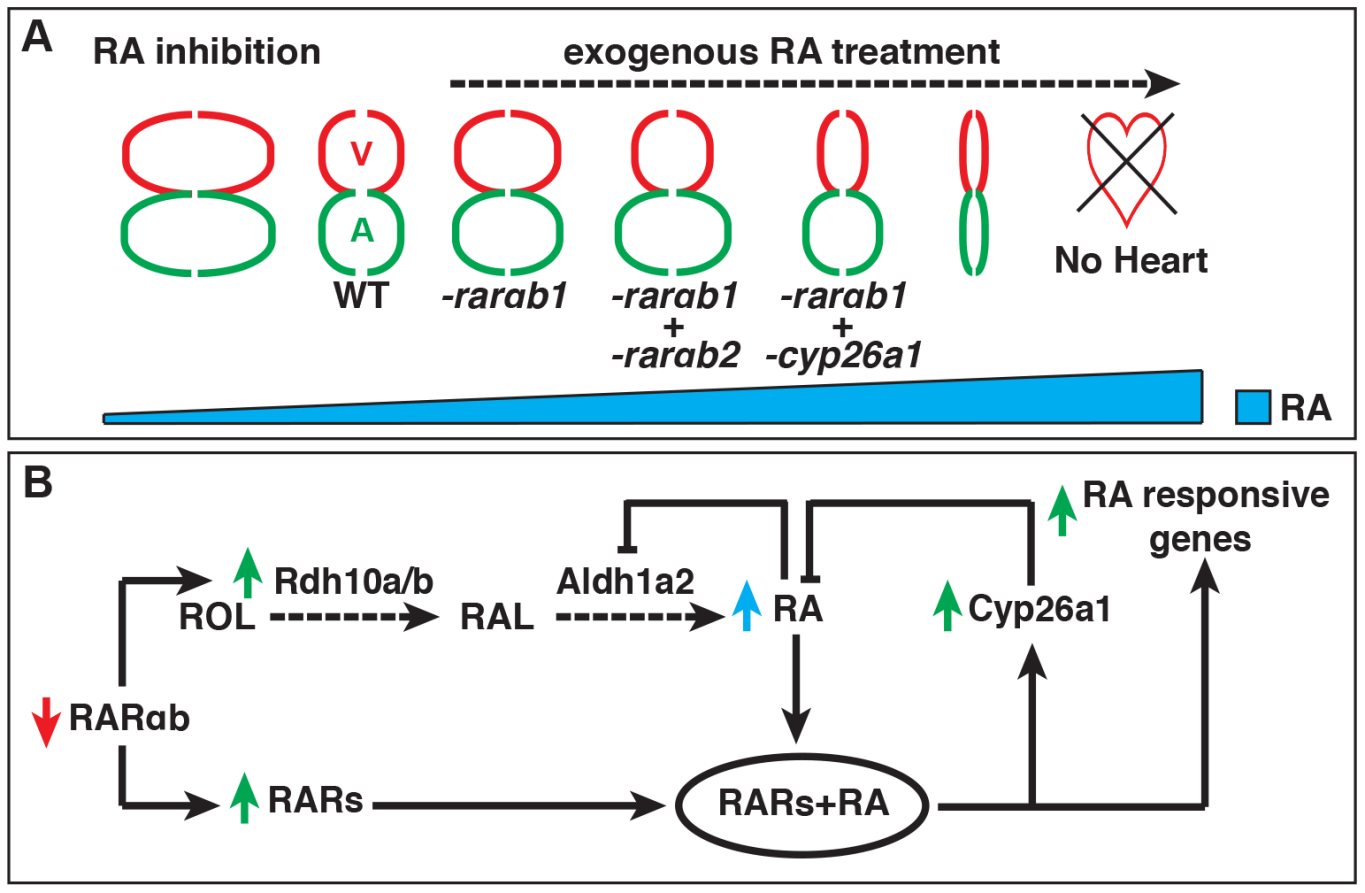
Models of the effects of RA signaling on heart patterning and the RA feedback mechanism. (A) Model depicting the consequences on atrial and ventricular CM specification at different levels of RA signaling. (B) Model of the previously unrecognized feedback mechanism that triggers increased RA signaling when depleting RARs. ROL = retinol. RAL = retinal. Red and green arrows indicate the effects on gene expression. Blue arrow indicates the effect on RA levels.

Together, our study supports a novel paradigm whereby RARαb depletion elicits a positive feedback mechanism that can result in teratogenic increases in RA signaling. Importantly, our work highlights that loss and gain of RA signaling can cause similar developmental defects. RA signaling is required to restrict CM specification 25,26, while high increases in RA signaling can eliminate CM specification (Figure 7A) 27. However, our present findings suggest that low increases in RA signaling, achieved when treating embryos with µM concentrations of RA or through RARαb depletion, can also promote increases in both atrial and ventricular CM specification (Figure 7A). As we found previously, modest, but slightly higher increases of RA signaling can promote atrial CM specification without significantly affecting ventricular CM specification 27, which is strikingly similar to what we found with concurrent depletion of the RARαb variants here (Figure 7A). Moreover, intermediate increases in RA signaling can inhibit ventricular CM specification, which is similar what we observed when concurrently depleting RARαb1 and Cyp26a1 (Figure 7A). It also appears that modulation of Hox activity downstream of both gain and loss RA signaling is at least partially responsible for the increases in CM specification, suggesting the hypothesis that the similar effects on CM number are actually due to opposite perturbations of anterior-posterior patterning within the ALPM. Therefore, our analysis corroborates and extends previous observations that there are differential effects on atrial and ventricular CM populations as there is a progressive increase from low to intermediate levels of RA signaling in the early embryo.

It is interesting that depletion of RARα homologs using MOs in zebrafish, presented in this study, and *Xenopus* 6 elicit similar phenotypic responses. In *Xenopus* embryos, RARα depletion alone results in loss of the MHB 6. While depletion of RARαb1 alone does not result in MHB defects in zebrafish embryos, we have found that RARαb1+Cyp26a1 deficient embryos completely lack the MHB. Taken together, these results suggest that the underlying consequences of increased RA signaling due to depletion of RARα homologs are likely conserved at least in *Xenopus* and zebrafish embryos, but that in *Xenopus* perhaps the role of Cyp26 enzymes in protecting the brain has been lost. Despite similarities in the phenotypes that both point to an increase in RA signaling in RARα and RARαb deficient *Xenopus* and zebrafish embryos, our results contrast with the model proposed by Koide et al. 6, which concluded that RARs are required to function as transcriptional repressors. Importantly, the tools used in the previous study, including dominant-negative RARs, transcriptional co-repressors, and inverse agonists, would not have allowed them to distinguish between a transcriptional de-repressive model and the positive feedback mechanism involving the production of excess RA supported here.

In addition to the phenotypic similarities when depleting RARα homologs in *Xenopus* and zebrafish, depletion of zebrafish RARαbs results in compensatory RAR expression similar to RARα depletion in mice 23, supporting the hypothesis that this feedback response to RARα deficiency is conserved in vertebrates. Importantly, the response to RAR depletion is likely different than complete ablation of RARs. RAR KO mice have not been reported to have compensatory increases in other RARs 11,12, suggesting that a complete loss of RAR expression may cause a breakdown of this feedback loop. However, when considering the probability that RAR expression would be completely lost vs. depleted, we postulate that insults resulting in depletion of RAR expression would be much more likely. Consistent with this idea, variable levels of RAR expression deficiency, which in the case of RARβ can be due to epigenetic silencing, is commonly observed in a variety of cancers 13.

Given the conserved feedback mechanisms already recognized that limit fluctuations in RA signaling in vertebrates 16,17,19,23, it seems logical that a conserved mechanism that senses RAR deficiency would also exist to prevent loss of RA signaling. We propose that this newly recognized positive feedback mechanism would be more suitable to prevent transient deficiency in RARs. As demonstrated here, persistent RARαb depletion can result in a hypervigilant response of RA signaling and RA-induced teratogenic defects. Overall, these data provide insight into a previously unappreciated RAR-dependent positive feedback mechanism (Figure 7B), which is active during development. Further elucidation of this RA signaling feedback mechanism may illuminate the etiology of poorly understood RA-insensitive cancers 13,23 and congenital defects 1,3.

## Materials And Methods

### Ethics Statement

All zebrafish husbandry and experiments were performed in accordance with protocols approved by the Institutional Animal Care and Use Committee (IACUC) of Cincinnati Children's Hospital Medical Center.

### Zebrafish Husbandry And Transgenic Lines

Zebrafish (*Danio rerio*) were raised and maintained as previously described 35. The following transgenic lines were used: *Tg(-5.1myl7:DsRed-NLS)* 36, *Tg(-5.1myl7:EGFP)^twu26^* 37, *Tg(12XRARE-ef1a:EGFP)^sk72^* 29,*Tg(β-actin:GDBD-RLBD)^cch1^* (was created using the Gateway/Tol2 system 38 and additional characterization is reported in 31), Tg(UAS:EGFP) 34, and *Tg(UAS:nfsB-mcherry)* 39.

### Ish

Whole-mount ISH was carried out using standard procedures 40. All probes except *rarαb1* (accession number: KF030797) and *rarαb2* were reported previously. *myl7* (formerly called *cmlc2*; ZDB-GENE-991019-3), *amhc* (ZDB-GENE-031112-1), *vmhc* (ZDB-GENE-991123-5), *nkx2.5* (ZDB-GENE-980526-321), *hand2* (ZDB-GENE-000511-1), *hoxb5a* (ZDB-GENE-980526-70), *hoxb5b* (ZDB-GENE-000823-6), *dhrs3a* (ZDB-GENE-040801-217), *cyp26a1* (ZDB-GENE-990415-44), *rarαb1/2* (which recognizes both isoforms and was formerly called *rarαb* 24; ZDB-GENE-980526-72), *rarαa* (ZDB-GENE-980526-284), *rarγa* (ZDB-GENE-980526-531), *rarγb* (ZDB-GENE-070314-1), *rdh10a* (ZDB-GENE-070112-2242), *tbx5a* (ZDB-GENE-030909-7), *eng2a* (ZDB-GENE-980526-167), *egr2b* (formerly called *krox20*; ZDB-GENE-980526-283), *egfp* (accession number: JQ064510.1), and *mcherry* (accession number: JN795134.1).

### Mo And Mrna Injections

The *rarαb1* MO (5′-TGCAGGTCATCCGTAATGCCCGATC) was designed to the 5′ UTR of *rarαb1*. Additional MOs targeting another region of the 5′ UTR and the donor splice junction, which saturated the available MO target sites, were also tried. However, injection of these MOs resulted in significant toxicity and were not able to be used for analysis. Sequences to the *rarαb2* and *hoxb5b* MOs were reported previously 8,26. The total amount of *rarαb1* MO injected was 16 ng. The total amount of *rarαb2* MO injected was 7 ng. The suboptimal doses used to test genetic interactions were half these concentrations. The amount of *hoxb5b* MO used was 0.25 ng. A cocktail of 4 ng *cyp26a1* MO1 (5′-TCTTATCATCCTTACCTTTTTCTTG) and 2 ng *cyp26a1* MO2 (5′-TAAAAATAATACACTACCTGCAAAC) produced a phenotype similar to *gir* mutants 17. Suboptimal doses used in experiments were 0.9 ng (*cyp26a1* MO1) and 0.45 ng of (*cyp26a1* MO2). For all injection experiments, 3 ng of *p53* MO were used to help suppress non-specific MO-induced cell death 41. For experiments, the total amount of MO injected was always kept constant by equilibrating the concentrations with Standard Control MO (Gene Tools).

Capped mRNA was made using a Message Machine Kit (Ambion). 150 pg of mRNA was used for over-expression of all mRNAs in all experiments.

### Cell Culture And Luciferase Assay

Luciferase reporter assays were performed in HEK 293 cells as previously described 29.

### Western Blot Analysis And Chip

Western blots were performed as previously described 29. Mouse monoclonal anti-myc antibody (Covance) was used for both Western blot analysis and ChIP experiments. The dynabeads (Invitrogen) ChIP protocol was adapted from the Dorsky Lab (University of Utah) ZFIN Protocol. qPCR was used to quantify the enrichment of the fragment containing the RARE (DR5) in embryos injected with the *myc-rarαb1* mRNA with respect to control uninjected embryos.

### Comparison Of Genomic Sequences

The genomic sequence flanking zebrafish *hoxb5b* (−8 to +8 kb) was compared with the corresponding region for *Hoxb5* in mouse using mVista. NHR SCAN was used to identify binding sites for nuclear receptor.

### Identification Of *Rarαb1* And Rt-Pcr

*Rarαb1* was identified by using BLAST against the zebrafish genome (Ensemble_V7) with the human and mouse RARα1 A domains. MacVector was used for sequences alignments. For RT-PCR, primer pairs were designed so that they specifically recognized *rarαb1* and *rarαb2* (Figure 1B). Primer sequences are available upon request.

### Cloning

The full-length coding sequence for *rarαb1* was cloned into pCS2p+. The *rarαb2*-pCS2p+ construct used for overexpression was reported previously 29. The myc tagged RARαb1 was made using the pCS2+MT vector. For *rarαb1 and rarαb2* probes, 536 base pairs (bps) of *rarαb1* and 443 bps of *rarαb2*, which include the 5′ untranslated region (UTR) and the specific A domains with no overlap, were cloned (Figure 1B). These fragments were cloned into pGEM-T easy (Promega).

### Qpcr

Total RNA was isolated from 25 embryos, homogenized in TRIzol (Ambion) and collected using Pure link RNA Micro Kit (In Vitrogen). 1 µg or 0.5 µg RNA was used for cDNA synthesis using the ThermoScript Reverse Transcriptase kit (Invitrogen). Quantitative real time PCR (qPCR) for *myl7*, *amhc*, *vmhc*, *nkx2.5*, *hand2*, *hoxb5b*, *hoxb5a*, *hoxb6b*, *dhrs3a*, *cyp26a1*, *aldhh1a2*, *rdh10a*, *rdh10b*, *rarαa*, *rarαb1*, *rarαb2*, *rarγa and rarγb*, *egfp* and *mcherry* was performed using standard PCR conditions in a Bio-Rad CFX PCR machine with Power SYBR Green PCR Master Mix (Applied Biosystems). Expression levels were standardized to *ef1*α expression and all the data were analyzed using the 2^−ΔΔCT^ Livak Method. All experiments were performed in a biological triplicate. Primer sequences are available upon request.

### Area And Length Measurements

Areas of *myl7*, *vmhc* and *amhc* expressing cells were measured using ImageJ and statistical analysis was performed as previously described 26. Length of *egfp* expression and distance between *hoxb5b* and *egr2b* were measured also using ImageJ and statistical analysis was performed as previously described.

### Imaging Of Zebrafish Heart And Cell Counting

Immunohistochemistry, cell counting and statistical analysis were done as previously described 26.

### Ra And Deab Treatment

RA and DEAB, treatment of embryos was done as previously described 26,27. Embryos that have been used for gene expression analysis at 8 somites were treated with 1 µM DEAB, an Aldh1a2 inhibitor, beginning at 40% epiboly or with 1 µM RA for 1 hr beginning at 40% epiboly. For analysis of the effects of low concentrations of RA on heart development, embryos were treated with 0.05 µM RA for 1 hr beginning at 40% epiboly and harvested at 48 hpf. For rescue experiments related to the heart phenotype of RARαb1 deficient embryos, embryos were treated with 0.025 µM DEAB beginning at 40% epiboly until 24 hpf. For rescue experiments related to the MHB in RARαb1+Cyp26a1 deficient embryos, embryos were treated with 0.25 µM DEAB.

### Statistical Analysis

To assess whether the means of two groups are statistically different from each other, we applied the Student's *t*-*test*. A *p* value of <0.05 was considered statistically significant.

## Supporting Information

Figure S1 **Comparison of RARαb1 and RARαb2 expression.** (A, D, G, J, M) *rarαb1* expression. (B, E, H, K, N) *rarαb2* expression. (C, F, I, L, O) *rarαb1/2* is a probe that recognizes both isoforms 24. Arrows in J and L indicate anterior ventral expression. Arrowheads in K and L indicate hindbrain and anterior spinal cord expression. Arrows in M and N indicate differences in the expression of the developing tail. In A–O, all views are lateral. In D–O, dorsal is to the right.
(TIF)

Figure S2 **RARαb1 deficient embryos have enlarged hearts at 72 hpf.** (A) Control sibling *Tg(-5.1myl7:GFP*)*^f2^* embryo. (C) RARαb1 deficient *Tg(-5.1myl7:GFP*)*^f2^* embryo. Arrow in C indicates pericardial edema with enlarged heart. (B, D) Higher magnification images of the fluorescent hearts of the *Tg(-5.1myl7:GFP*)*^f2^* control sibling and RARαb1 deficient *Tg(-5.1myl7:GFP*)^f2^ embryos in A and C. Images are lateral views with dorsal up and anterior right.
(TIF)

Figure S3 **RARαb1 deficient embryos have enlarged hearts with increased CM number at 55 hpf.** (A, B) Hearts from control sibling and RARαb1 deficient *Tg(-5.1myl7:DsRed-NLS)^f2^* embryos at 55 hpf. Images are frontal views. Red indicates ventricle. Green indicates atrium. (C) Mean CM number at 48 hpf.
(TIF)

Figure S4 **Specificity controls for the translation blocking *rarαb1* MO.** (A–C) Control sibling, RARαb1 deficient, and RARαb1 deficient+*rarαb1* mRNA injected embryos. Images are lateral views with anterior right at 48 hpf. Red outline indicates ventricles. Green outline indicates atria. Arrow in B indicates edema often found in RARαb1 deficient embryos, which is not found in RARαb1 deficient+*rarαb1* mRNA injected embryos (C). (D) qPCR for CM differentiation marker genes at 48 hpf in control sibling, RARαb1 deficient, RARαb1 deficient embryos+*rarαb1* mRNA, and RARαb1 deficient embryos+*kaede* (control) mRNA injected embryos at 48 hpf. Pound sign indicates a statistically significant difference compared to RARαb1 deficient and RARαb1 deficient embryos+*kaede* (control) mRNA injected embryos (p<0.05).
(TIF)

Figure S5 **RARs can directly bind the RA response element (RARE) in the zebrafish *hoxb5b* regulatory region.** (A) mVista sequence alignment of mouse *Hoxb5* and zebrafish *hoxb5b* genomic regions. Purple boxes represent exons. Light blue boxes indicates 5′ and 3′ UTR. Peaks represents levels of sequence identity in a 50 bp window. Purple peaks are conserved regions in exons. Light blue peaks are conserved regions in 5′ UTR. Pink peaks are conserved non-coding sequences. Arrow indicates the presence of a RARE in the conserved sequence between 4 kb and 4.5 kb identified previously 42, which we confirmed using the NHR SCAN database. (B) Sequence conservation (red) between mouse and zebrafish DR5 RARE. (C) Western blot for myc-tagged RARαb1. (D) ChIP from control sibling and *myc-rarαb1* mRNA injected embryos. Negative control *amhc* primers did not detect any enrichment (data not shown).
(TIF)

Figure S6 **A suboptimal dose of *hoxb5b* MO does not affect CM cell number at 48 hpf.** (A, B) Hearts from control sibling and Hoxb5b deficient *Tg(-5.1myl7:DsRed-NLS)^f2^* embryos at 48 hpf. Images are frontal views. Red indicates ventricle. Green indicates atrium. (C) Mean CM number at 48 hpf.
(TIF)

Figure S7 **RARαb1 and RARαb2 function partially redundantly to promote forelimb development.** (A–C) Control sibling, RARαb1 deficient, and RARαb2 deficient embryos. Images in A–C are dorsal views with anterior to the left. Arrows in B indicate smaller forelimbs. (D) Percentage of control sibling (n = 20), RARαb1 deficient (n = 20), and RARαb2 deficient (n = 20) embryos with normal, small or no forelimbs. An optimal dose of the *rarαb1* and *rarαb2* MOs was used for experiments in D. (E) Percentage of embryos with normal, small, or no forelimbs after injection with a suboptimal dose of *rarαb1* MO (n = 28), a suboptimal dose of *rarαb2* MO (n = 26), and co-injected with suboptimal doses of the *rarαb1* and *rarαb2* MOs (n = 17). (F–H) ISH of *tbx5a*, a forelimb marker, in control sibling, RARαb1 deficient, RARαb2 deficient embryos. Arrows in F–H indicate *tbx5a* expression the LPM. (I) Areas of the amount of cells expressing the *tbx5a* at 24 hpf.
(TIF)

Figure S8 **RARαb2 deficient embryos do not have enlarged hearts.** (A, B) Hearts from control sibling and RARαb2 deficient *Tg(-5.1myl7:DsRed-NLS)* embryos at 48 hpf. Images are frontal views. Red indicates ventricle. Green indicates atrium. (C) Mean CM number from the hearts of control sibling and RARαb2 deficient *Tg(-5.1myl7:DsRed-NLS)* embryos at 48 hpf. (D) qPCR for CM marker gene expression in control sibling and RARαb2 deficient embryos at 48 hpf. We do find a modest decrease in CM number (C) and *myl7* expression (D), which is likely due to a very modest amount of MO-induced toxicity.
(TIF)

Figure S9 **Patterning of the spinal cord is affected in the RARαb1+RARαb2 deficient embryos.** (A–D) *Hoxb5a* (spinal cord) and *egr2b* (rhombomeres 3+5) expression in control (n = 32), RARαb1 deficient (n = 23), RARαb2 deficient (n = 16), and RARαb1+RARαb2 deficient embryos (n = 19). (E) Measurements of the distance in arbitrary units (AU) between *hoxb5a* and *egr2b* expression. Expression of *hoxb5a* in the spinal cord is expanded rostrally. The rostral expansion of *hoxb5a* in RARαb1 deficient embryos trends similarly as RARαb2 deficient and RARαb1+RARαb2 deficient embryos, but it is not statistically significant (p = 0.06).
(TIF)

Figure S10 **RARαb1 and RARαb2 function partially redundantly to promote proper heart development.** (A–D) Control sibling, RARαb1 deficient (suboptimal dose), RARαb2 deficient (suboptimal dose), and RARαb1+RARαb2 (suboptimal doses) deficient embryos at the 72 hpf. Arrow in D indicates pericardial edema and the enlarged heart.
(TIF)

Figure S11 ***Rarαb1* and *rarαb2* mRNA overexpression do not significantly affect RA responsive genes. (A–I) ISH for the RA responsive genes *cyp26a1*, *dhrs3a*, and *hoxb5b* at 8 s.** (A, D, G) Control sibling, (B, E, H) *rarαb1* mRNA, and (C, F, I) *rarαb2* mRNA injected embryos. Injection of either *rarαb* mRNA did not inhibit RA responsive gene expression. Images in A–C are lateral views with anterior up and dorsal right. Images in D–I are dorsal views with anterior up. (J) qPCR for RA responsive genes *cyp26a1*, *hoxb5a*, *hoxb8b*, *dhrs3a* at 8 s. (K) Mean CM number from control sibling, *rarαb1* mRNA, and *rarαb2* mRNA injected *Tg(-5.1myl7:DsRed-NLS)^f2^* embryos. (L) Transfection of HEK 293 cells with DNA for the zebrafish *rarαb1* and *rarαb2* and pGL3-12XRARE-*ef1α*:*renilla luciferase* vector with and without RA treatment. Fold difference in luminescence is indicated in arbitrary units (AU) and reflects the ratio of renilla luciferase (RL) to firefly (FL) luciferase.
(TIF)

Figure S12 ***Rar* expression in RARαb1, RARαb2, or RARαb1+2 deficient embryos.** (A, B) ISH for *rarαb1* in RARαb2 deficient embryos. (C, D) ISH for *rarαb2* in *RARαb1* deficient embryos. (E, F) ISH for *rarαa2* in RARαb1+2 deficient embryos. (G, H) ISH for *rarγa* in RARαb1+2 deficient embryos. (I, L) ISH for *rarγb* in *Rarαb1+2* deficient embryos. *rar* expression is often expanded in the tailbud region of embryos deficient for the other RAR homologs, while additional regions also appear to have increased or low levels of ectopic expression. All views are lateral with dorsal right at 8 s. Arrows in A–H indicate distance of expression in the tail. Arrowheads in F, H, L indicate regions of increased or ectopic expression.
(TIF)

Figure S13 ***Rdh10a* expression in RARαb1+2 deficient embryos.** (A–D) ISH for *rdh10a in* RARαb1+2 deficient embryos at 8 somites. (A, B) Lateral views with dorsal right. (C, D) Dorsal views with anterior up. Brackets indicate expansion of *rdh10a* in the ALPM. Arrow indicates increased expression in the somites.
(TIF)

Figure S14 **Characterization of *cyp26a1* splice-blocking MOs used in experiments.** (A) Schematic of the *cyp26a1* locus and the intron-exon boundaries targeted by the different *cyp26a1* MOs. Blue bar indicates MO1. Red bar indicates MO2. MO1 primarily causes usage of two in-frame cryptic splice sites. Dashed lines indicate the alternate introns cause by the cryptic splices induced from MO1. MO2 causes the introduction of a premature stop codon (red X). (B) RT-PCR for the WT *cyp26a1* transcripts and alternate transcripts induced from the different MOs. U and L indicate bands depicted in A. (C) Control sibling embryo. (D) Embryos injected with cocktail of *cyp26a1* MO1+2. Co-injection of *cyp26a1* MO1 and MO2 causes a phenotype equivalent to or stronger than the *cyp26a1/giraffe (gir)* mutant (E). Injection of the individual MOs causes the phenotypes consistent with *cyp26a1* loss of function at low frequency (data not shown). A suboptimal dose of the *cyp26a1* MO cocktail was used for functional interaction experiments with RARαb1 (Figure 4). Arrows in D and E indicate shortened tail. Views in C–E are lateral with anterior right.
(TIF)

Figure S15 **Characterization of the novel transgenic RA sensorline.** (A) Schematic of the RAR domains and the Gal4 DNA binding domain (GDBD)/RARαb ligand binding domain (RLBD) fusion protein. Grey indicates the GDBD. Yellow indicates the RLBD. D is a linker domain and F is a domain with unknown function (as in Figure 1). (B, C) Schematics representing the GDBD-RLBD fusion acting on the *Gal4-UAS:EGFP* transgene. The GDBD-RLBD is expressed under the β-actin promoter. (B) In the absence of RA, *egfp* is not expressed. (C) In the presence of RA (red triangles), the GDBD-RLBD is able to promoted *egfp* (UAS responsive gene) transcription. (D–G) *Tg(β-actin:GDBD-RLB)*;*Tg(UAS:nfsB-mcherry)* embryos are responsive to RA treatment. ISH for *mcherry*. Equivalent results were found when the *Tg(β-actin:GDBD-RLB)* line was crossed to *Tg(UAS:EGFP)* fish (data not shown) as were used for experiments in Figure 5. More detailed characterization of the stable transgenic RA sensor lines is reported in 31. (D, E) Lateral views with dorsal right. (F, G) Dorsal views. In images D–G anterior is up.
(TIF)

## References

[1] Niederreither K, Dolle P (2008) Retinoic acid in development: towards an integrated view. Nature reviews Genetics 9: 541–553. 10.1038/nrg2340 18542081

[2] Rhinn M, Dolle P (2012) Retinoic acid signalling during development. Development 139: 843–858. 22318625 10.1242/dev.065938

[3] Lammer EJ, Chen DT, Hoar RM, Agnish ND, Benke PJ, et al (1985) Retinoic acid embryopathy. N Engl J Med 313: 837–841. 3162101 10.1056/NEJM198510033131401

[4] Rizzo R, Lammer EJ, Parano E, Pavone L, Argyle JC (1991) Limb reduction defects in humans associated with prenatal isotretinoin exposure. Teratology 44: 599–604. 1805430 10.1002/tera.1420440602

[5] Bastien J, Rochette-Egly C (2004) Nuclear retinoid receptors and the transcription of retinoid-target genes. Gene 328: 1–16. 15019979 10.1016/j.gene.2003.12.005

[6] Koide T, Downes M, Chandraratna RA, Blumberg B, Umesono K (2001) Active repression of RAR signaling is required for head formation. Genes & development 15: 2111–2121. 11511542 10.1101/gad.908801 PMC312762

[7] Li P, Pashmforoush M, Sucov HM (2010) Retinoic acid regulates differentiation of the secondary heart field and TGFbeta-mediated outflow tract septation. Developmental Cell 18: 480–485. 20230754 10.1016/j.devcel.2009.12.019 PMC2841063

[8] Linville A, Radtke K, Waxman JS, Yelon D, Schilling TF (2009) Combinatorial roles for zebrafish retinoic acid receptors in the hindbrain, limbs and pharyngeal arches. Developmental Biology 325: 60–70. 18929555 10.1016/j.ydbio.2008.09.022 PMC3045866

[9] Lohnes D, Mark M, Mendelsohn C, Dolle P, Dierich A, et al (1994) Function of the retinoic acid receptors (RARs) during development (I). Craniofacial and skeletal abnormalities in RAR double mutants. Development 120: 2723–2748. 7607067 10.1242/dev.120.10.2723

[10] Mendelsohn C, Lohnes D, Decimo D, Lufkin T, LeMeur M, et al (1994) Function of the retinoic acid receptors (RARs) during development (II). Multiple abnormalities at various stages of organogenesis in RAR double mutants. Development 120: 2749–2771. 7607068 10.1242/dev.120.10.2749

[11] Lohnes D, Kastner P, Dierich A, Mark M, LeMeur M, et al (1993) Function of retinoic acid receptor gamma in the mouse. Cell 73: 643–658. 8388780 10.1016/0092-8674(93)90246-m

[12] Lufkin T, Lohnes D, Mark M, Dierich A, Gorry P, et al (1993) High postnatal lethality and testis degeneration in retinoic acid receptor alpha mutant mice. Proceedings of the National Academy of Sciences of the United States of America 90: 7225–7229. 8394014 10.1073/pnas.90.15.7225 PMC47109

[13] Soprano DR, Qin P, Soprano KJ (2004) Retinoic acid receptors and cancers. Annu Rev Nutr 24: 201–221. 15189119 10.1146/annurev.nutr.24.012003.132407

[14] Sandell LL, Lynn ML, Inman KE, McDowell W, Trainor PA (2012) RDH10 oxidation of Vitamin A is a critical control step in synthesis of retinoic acid during mouse embryogenesis. PLoS One 7: e30698. 22319578 10.1371/journal.pone.0030698 PMC3271098

[15] Sandell LL, Sanderson BW, Moiseyev G, Johnson T, Mushegian A, et al (2007) RDH10 is essential for synthesis of embryonic retinoic acid and is required for limb, craniofacial, and organ development. Genes & development 21: 1113–1124. 17473173 10.1101/gad.1533407 PMC1855236

[16] Dobbs-McAuliffe B, Zhao Q, Linney E (2004) Feedback mechanisms regulate retinoic acid production and degradation in the zebrafish embryo. Mechanisms of Development 121: 339–350. 15110044 10.1016/j.mod.2004.02.008

[17] Emoto Y, Wada H, Okamoto H, Kudo A, Imai Y (2005) Retinoic acid-metabolizing enzyme Cyp26a1 is essential for determining territories of hindbrain and spinal cord in zebrafish. Developmental Biology 278: 415–427. 15680360 10.1016/j.ydbio.2004.11.023

[18] Niederreither K, McCaffery P, Drager UC, Chambon P, Dolle P (1997) Restricted expression and retinoic acid-induced downregulation of the retinaldehyde dehydrogenase type 2 (RALDH-2) gene during mouse development. Mechanisms of Development 62: 67–78. 9106168 10.1016/s0925-4773(96)00653-3

[19] Cai AQ, Radtke K, Linville A, Lander AD, Nie Q, et al (2012) Cellular retinoic acid-binding proteins are essential for hindbrain patterning and signal robustness in zebrafish. Development 139: 2150–2155. 22619388 10.1242/dev.077065 PMC3357909

[20] de The H, Vivanco-Ruiz MM, Tiollais P, Stunnenberg H, Dejean A (1990) Identification of a retinoic acid responsive element in the retinoic acid receptor beta gene. Nature 343: 177–180. 2153268 10.1038/343177a0

[21] Hoffmann B, Lehmann JM, Zhang XK, Hermann T, Husmann M, et al (1990) A retinoic acid receptor-specific element controls the retinoic acid receptor-beta promoter. Molecular Endocrinology 4: 1727–1736. 2177841 10.1210/mend-4-11-1727

[22] Leroy P, Nakshatri H, Chambon P (1991) Mouse retinoic acid receptor alpha 2 isoform is transcribed from a promoter that contains a retinoic acid response element. Proceedings of the National Academy of Sciences of the United States of America 88: 10138–10142. 1658797 10.1073/pnas.88.22.10138 PMC52883

[23] Manshouri T, Yang Y, Lin H, Stass SA, Glassman AB, et al (1997) Downregulation of RAR alpha in mice by antisense transgene leads to a compensatory increase in RAR beta and RAR gamma and development of lymphoma. Blood 89: 2507–2515. 9116296

[24] Waxman JS, Yelon D (2007) Comparison of the expression patterns of newly identified zebrafish retinoic acid and retinoid X receptors. Developmental dynamics : an official publication of the American Association of Anatomists 236: 587–595. 17195188 10.1002/dvdy.21049

[25] Keegan BR, Feldman JL, Begemann G, Ingham PW, Yelon D (2005) Retinoic acid signaling restricts the cardiac progenitor pool. Science 307: 247–249. 15653502 10.1126/science.1101573

[26] Waxman JS, Keegan BR, Roberts RW, Poss KD, Yelon D (2008) Hoxb5b acts downstream of retinoic acid signaling in the forelimb field to restrict heart field potential in zebrafish. Developmental Cell 15: 923–934. 19081079 10.1016/j.devcel.2008.09.009 PMC2752051

[27] Waxman JS, Yelon D (2009) Increased Hox activity mimics the teratogenic effects of excess retinoic acid signaling. Developmental dynamics : an official publication of the American Association of Anatomists 238: 1207–1213. 19384962 10.1002/dvdy.21951 PMC2739864

[28] He X, Yan YL, Eberhart JK, Herpin A, Wagner TU, et al (2011) miR-196 regulates axial patterning and pectoral appendage initiation. Developmental Biology 357: 463–477. 21787766 10.1016/j.ydbio.2011.07.014 PMC3164755

[29] Waxman JS, Yelon D (2011) Zebrafish retinoic acid receptors function as context-dependent transcriptional activators. Developmental Biology 352: 128–140. 21276787 10.1016/j.ydbio.2011.01.022 PMC3207040

[30] Martin BL, Kimelman D (2010) Brachyury establishes the embryonic mesodermal progenitor niche. Genes & development 24: 2778–2783. 21159819 10.1101/gad.1962910 PMC3003196

[31] Mandal A, Rydeen A, Anderson J, Sorrell MR, Zygmunt T, et al (2013) Transgenic retinoic acid sensor lines in zebrafish indicate regions of available embryonic retinoic acid. Developmental dynamics : an official publication of the American Association of Anatomists 10.1002/dvdy.23987 PMC3771353 23703807

[32] Tiefenbach J, Moll PR, Nelson MR, Hu C, Baev L, et al (2010) A live zebrafish-based screening system for human nuclear receptor ligand and cofactor discovery. PLoS One 5: e9797. 20339547 10.1371/journal.pone.0009797 PMC2842432

[33] Allenby G, Bocquel MT, Saunders M, Kazmer S, Speck J, et al (1993) Retinoic acid receptors and retinoid X receptors: interactions with endogenous retinoic acids. Proceedings of the National Academy of Sciences of the United States of America 90: 30–34. 8380496 10.1073/pnas.90.1.30 PMC45593

[34] Asakawa K, Suster ML, Mizusawa K, Nagayoshi S, Kotani T, et al (2008) Genetic dissection of neural circuits by Tol2 transposon-mediated Gal4 gene and enhancer trapping in zebrafish. Proceedings of the National Academy of Sciences of the United States of America 105: 1255–1260. 18202183 10.1073/pnas.0704963105 PMC2234125

[35] Westerfield M (1993) The zebrafish book : a guide for the laboratory use of zebrafish (Brachydanio rerio). Eugene, OR: M. Westerfield. 1 v. (unpaged)

[36] Mably JD, Mohideen MA, Burns CG, Chen JN, Fishman MC (2003) heart of glass regulates the concentric growth of the heart in zebrafish. Current biology : CB 13: 2138–2147. 14680629 10.1016/j.cub.2003.11.055

[37] Huang CJ, Tu CT, Hsiao CD, Hsieh FJ, Tsai HJ (2003) Germ-line transmission of a myocardium-specific GFP transgene reveals critical regulatory elements in the cardiac myosin light chain 2 promoter of zebrafish. Developmental dynamics : an official publication of the American Association of Anatomists 228: 30–40. 12950077 10.1002/dvdy.10356

[38] Kwan KM, Fujimoto E, Grabher C, Mangum BD, Hardy ME, et al (2007) The Tol2kit: a multisite gateway-based construction kit for Tol2 transposon transgenesis constructs. Developmental Dynamics 236: 3088–3099. 17937395 10.1002/dvdy.21343

[39] Davison JM, Akitake CM, Goll MG, Rhee JM, Gosse N, et al (2007) Transactivation from Gal4-VP16 transgenic insertions for tissue-specific cell labeling and ablation in zebrafish. Developmental Biology 304: 811–824. 17335798 10.1016/j.ydbio.2007.01.033 PMC3470427

[40] Oxtoby E, Jowett T (1993) Cloning of the zebrafish krox-20 gene (krx-20) and its expression during hindbrain development. Nucleic Acids Res 21: 1087–1095. 8464695 10.1093/nar/21.5.1087 PMC309267

[41] Robu ME, Larson JD, Nasevicius A, Beiraghi S, Brenner C, et al (2007) p53 activation by knockdown technologies. PLoS genetics 3: e78. 17530925 10.1371/journal.pgen.0030078 PMC1877875

[42] Jarinova O, Hatch G, Poitras L, Prudhomme C, Grzyb M, et al (2008) Functional resolution of duplicated hoxb5 genes in teleosts. Development 135: 3543–3553. 18832391 10.1242/dev.025817

