# Supplementary figures and images for: Depletion of Retinoic Acid Receptors Initiates a Novel Positive Feedback Mechanism that Promotes Teratogenic Increases in Retinoic Acid
Source: PLoS Genet. 2013 Aug 8;9(8):e1003689. doi: 10.1371/journal.pgen.1003689 (PMC3750112; doi:10.1371/journal.pgen.1003689)

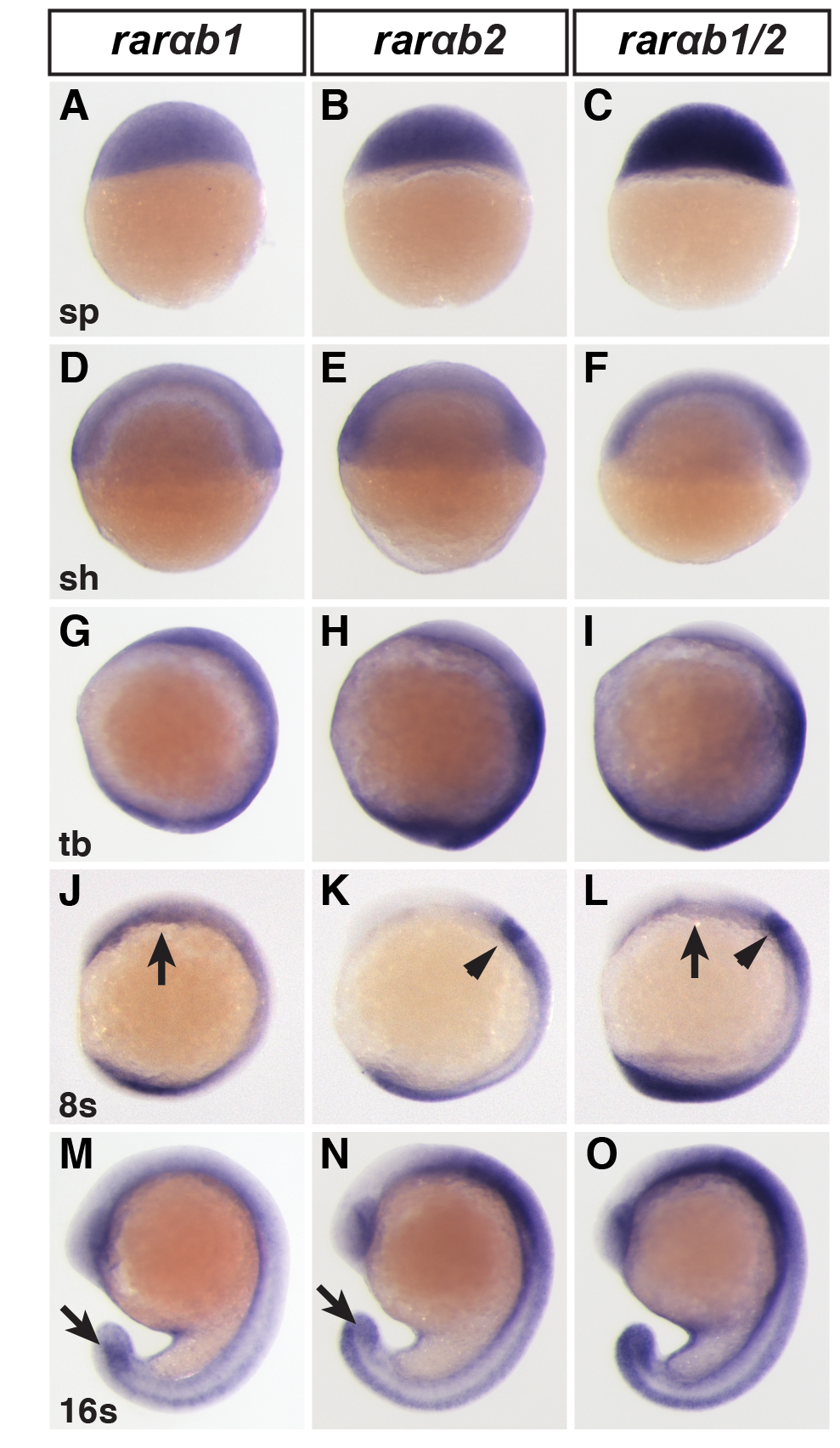

Supplement: Figure S1 — Comparison of RARαb1 and RARαb2 expression. (A, D, G, J, M) rarαb1 expression. (B, E, H, K, N) rarαb2 expression. (C, F, I, L, O) rarαb1/2 is a probe that recognizes both isoforms 24. Arrows in J and L indicate anterior ventral expression. Arrowheads in K and L indicate hindbrain and anterior spinal cord expression. Arrows in M and N indicate differences in the expression of the developing tail. In A–O, all views are lateral. In D–O, dorsal is to the right. (TIF) [file pgen.1003689.s001.tif]

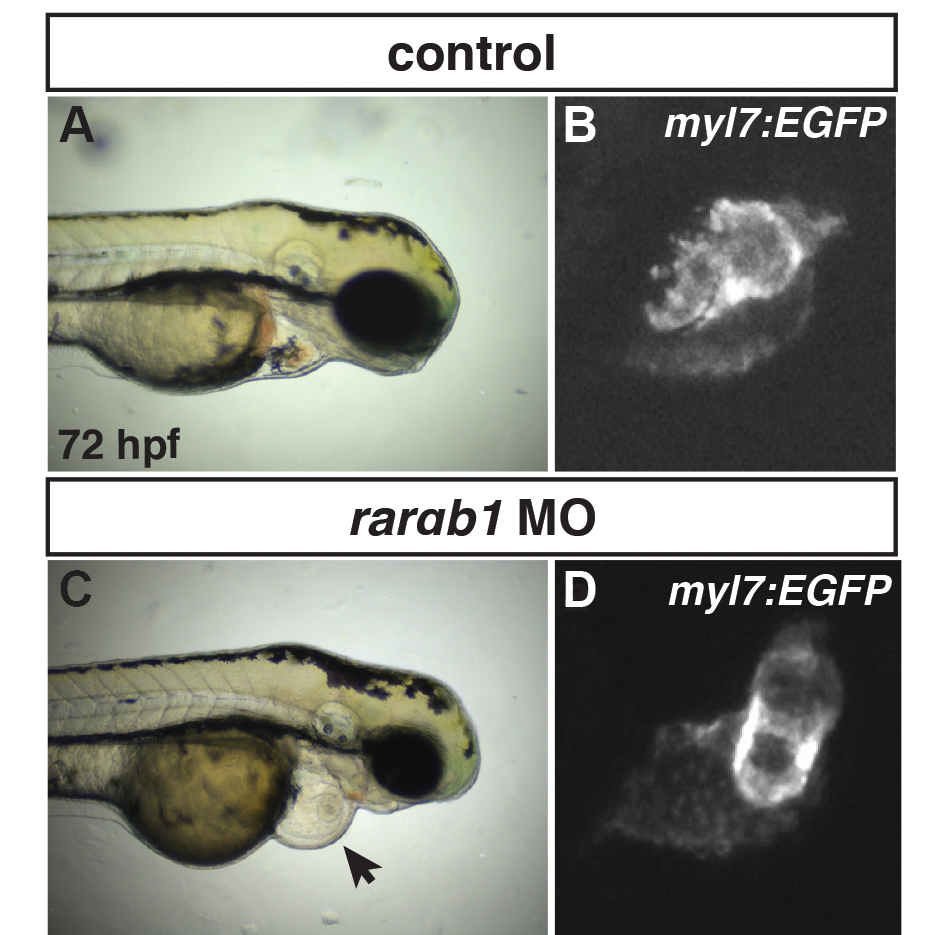

Supplement: Figure S2 — RARαb1 deficient embryos have enlarged hearts at 72 hpf. (A) Control sibling Tg(-5.1myl7:GFP)f2 embryo. (C) RARαb1 deficient Tg(-5.1myl7:GFP)f2 embryo. Arrow in C indicates pericardial edema with enlarged heart. (B, D) Higher magnification images of the fluorescent hearts of the Tg(-5.1myl7:GFP)f2 control sibling and RARαb1 deficient Tg(-5.1myl7:GFP)f2 embryos in A and C. Images are lateral views with dorsal up and anterior right. (TIF) [file pgen.1003689.s002.tif]

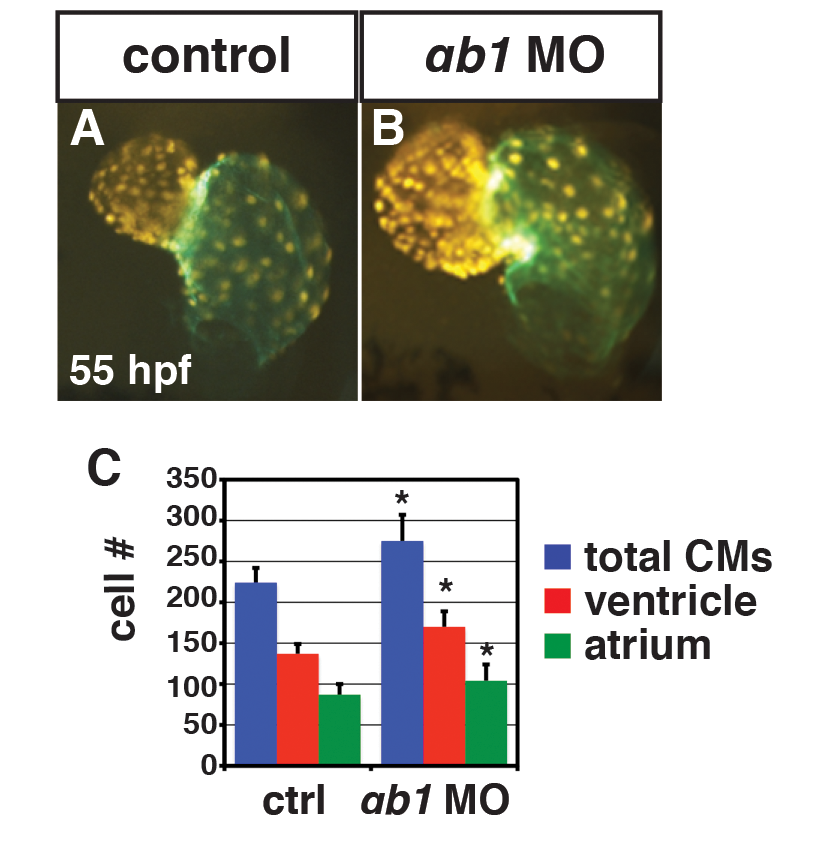

Supplement: Figure S3 — RARαb1 deficient embryos have enlarged hearts with increased CM number at 55 hpf. (A, B) Hearts from control sibling and RARαb1 deficient Tg(-5.1myl7:DsRed-NLS)f2 embryos at 55 hpf. Images are frontal views. Red indicates ventricle. Green indicates atrium. (C) Mean CM number at 48 hpf. (TIF) [file pgen.1003689.s003.tif]

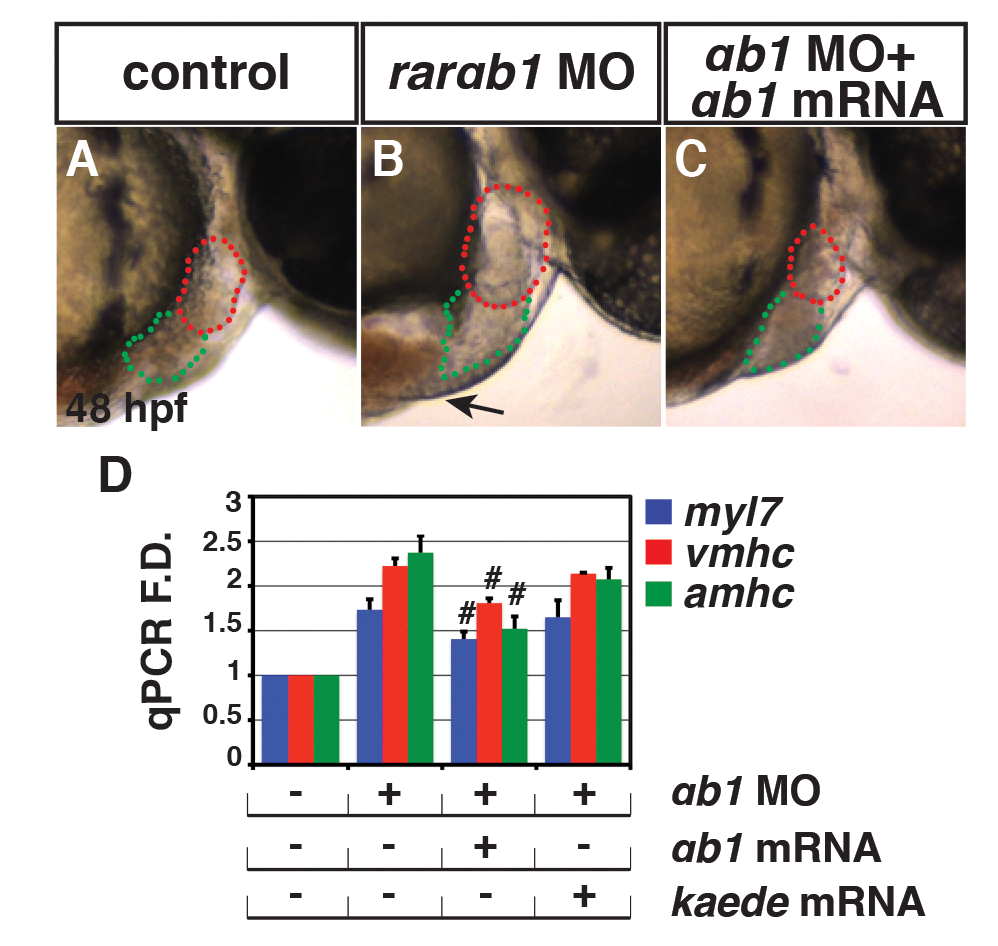

Supplement: Figure S4 — Specificity controls for the translation blocking rarαb1 MO. (A–C) Control sibling, RARαb1 deficient, and RARαb1 deficient+rarαb1 mRNA injected embryos. Images are lateral views with anterior right at 48 hpf. Red outline indicates ventricles. Green outline indicates atria. Arrow in B indicates edema often found in RARαb1 deficient embryos, which is not found in RARαb1 deficient+rarαb1 mRNA injected embryos (C). (D) qPCR for CM differentiation marker genes at 48 hpf in control sibling, RARαb1 deficient, RARαb1 deficient embryos+rarαb1 mRNA, and RARαb1 deficient embryos+kaede (control) mRNA injected embryos at 48 hpf. Pound sign indicates a statistically significant difference compared to RARαb1 deficient and RARαb1 deficient embryos+kaede (control) mRNA injected embryos (p<0.05). (TIF) [file pgen.1003689.s004.tif]

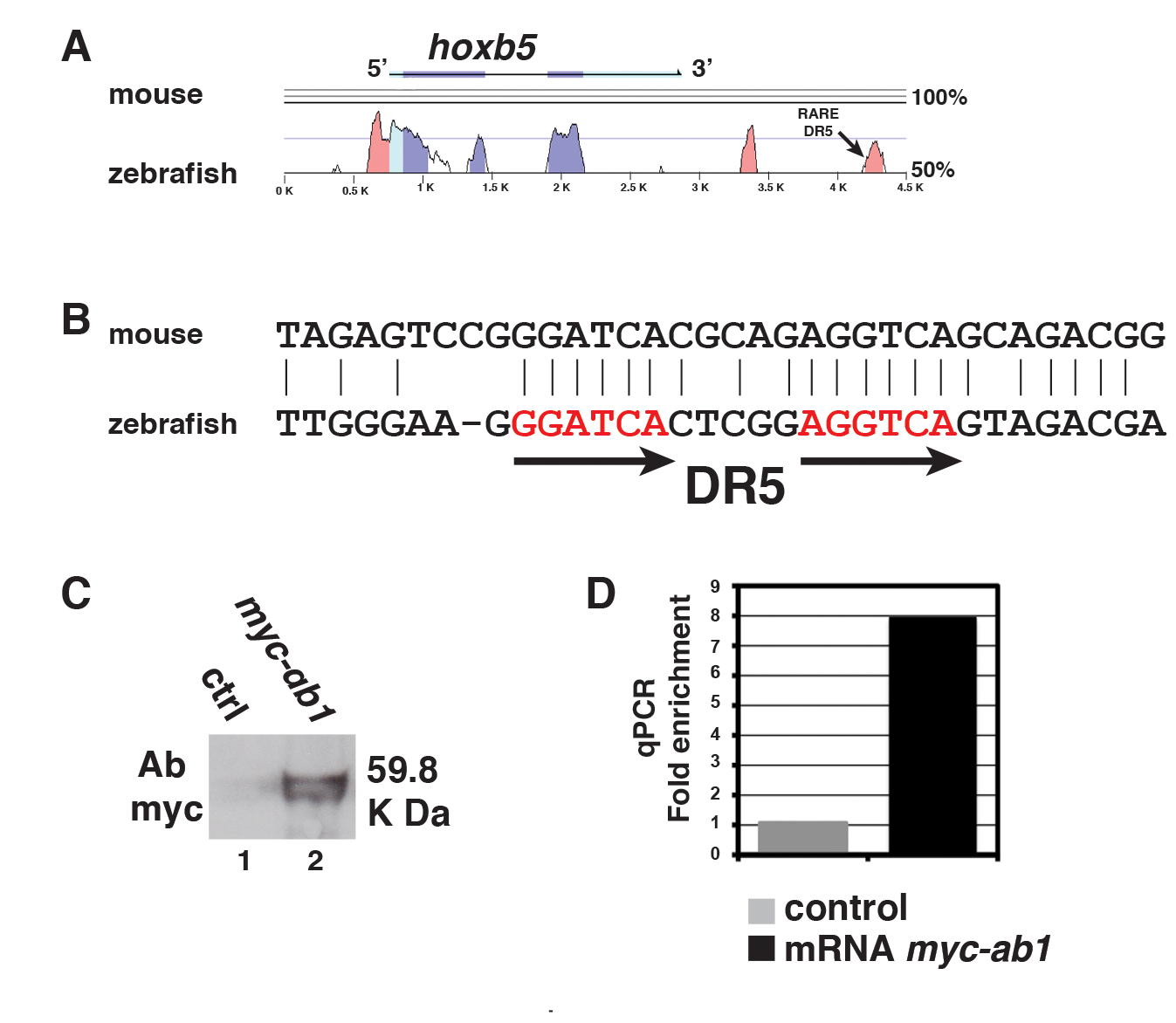

Supplement: Figure S5 — RARs can directly bind the RA response element (RARE) in the zebrafish hoxb5b regulatory region. (A) mVista sequence alignment of mouse Hoxb5 and zebrafish hoxb5b genomic regions. Purple boxes represent exons. Light blue boxes indicates 5′ and 3′ UTR. Peaks represents levels of sequence identity in a 50 bp window. Purple peaks are conserved regions in exons. Light blue peaks are conserved regions in 5′ UTR. Pink peaks are conserved non-coding sequences. Arrow indicates the presence of a RARE in the conserved sequence between 4 kb and 4.5 kb identified previously 42, which we confirmed using the NHR SCAN database. (B) Sequence conservation (red) between mouse and zebrafish DR5 RARE. (C) Western blot for myc-tagged RARαb1. (D) ChIP from control sibling and myc-rarαb1 mRNA injected embryos. Negative control amhc primers did not detect any enrichment (data not shown). (TIF) [file pgen.1003689.s005.tif]

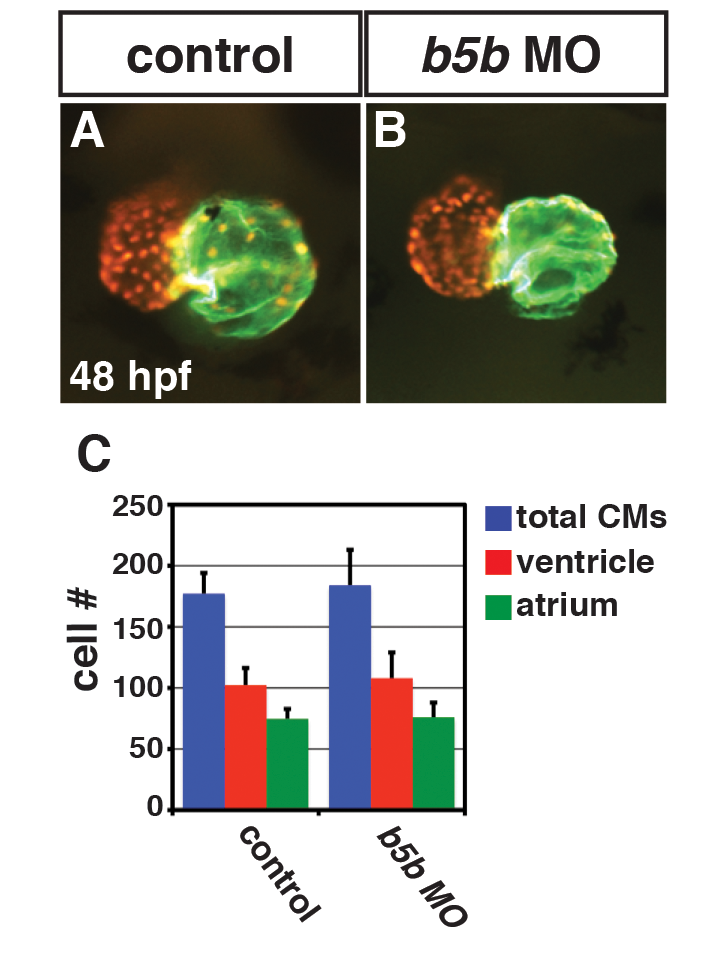

Supplement: Figure S6 — A suboptimal dose of hoxb5b MO does not affect CM cell number at 48 hpf. (A, B) Hearts from control sibling and Hoxb5b deficient Tg(-5.1myl7:DsRed-NLS)f2 embryos at 48 hpf. Images are frontal views. Red indicates ventricle. Green indicates atrium. (C) Mean CM number at 48 hpf. (TIF) [file pgen.1003689.s006.tif]

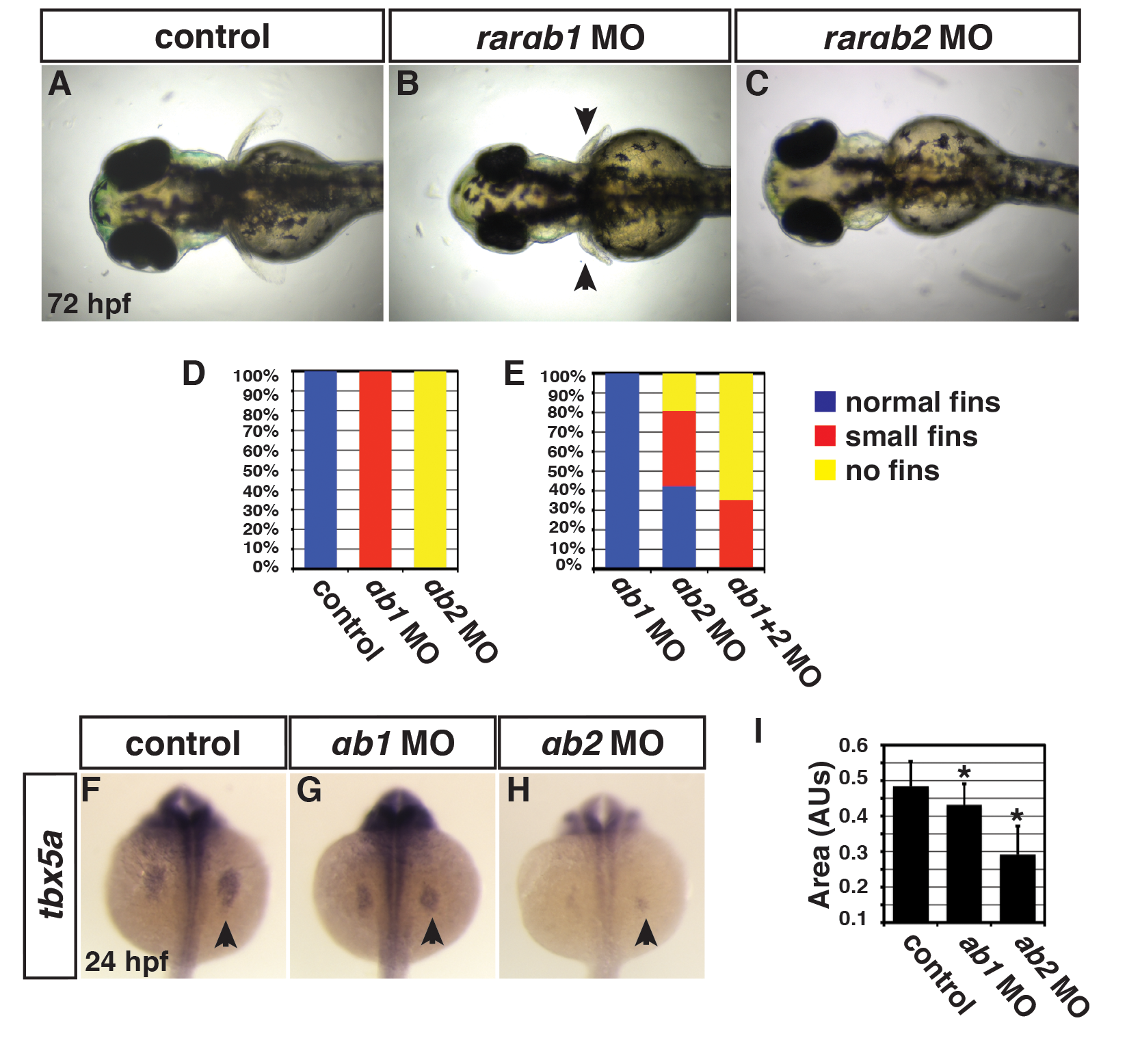

Supplement: Figure S7 — RARαb1 and RARαb2 function partially redundantly to promote forelimb development. (A–C) Control sibling, RARαb1 deficient, and RARαb2 deficient embryos. Images in A–C are dorsal views with anterior to the left. Arrows in B indicate smaller forelimbs. (D) Percentage of control sibling (n = 20), RARαb1 deficient (n = 20), and RARαb2 deficient (n = 20) embryos with normal, small or no forelimbs. An optimal dose of the rarαb1 and rarαb2 MOs was used for experiments in D. (E) Percentage of embryos with normal, small, or no forelimbs after injection with a suboptimal dose of rarαb1 MO (n = 28), a suboptimal dose of rarαb2 MO (n = 26), and co-injected with suboptimal doses of the rarαb1 and rarαb2 MOs (n = 17). (F–H) ISH of tbx5a, a forelimb marker, in control sibling, RARαb1 deficient, RARαb2 deficient embryos. Arrows in F–H indicate tbx5a expression the LPM. (I) Areas of the amount of cells expressing the tbx5a at 24 hpf. (TIF) [file pgen.1003689.s007.tif]

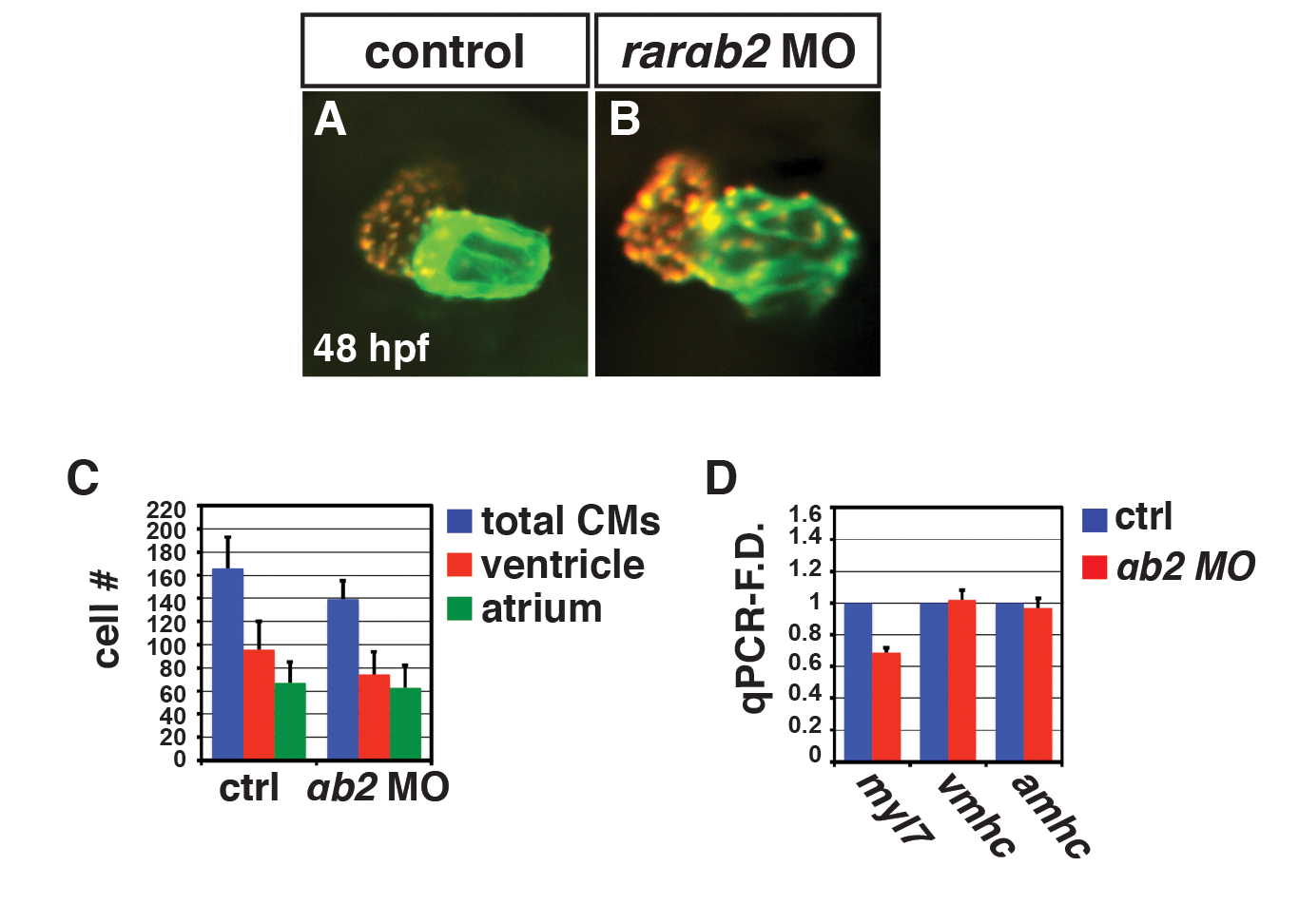

Supplement: Figure S8 — RARαb2 deficient embryos do not have enlarged hearts. (A, B) Hearts from control sibling and RARαb2 deficient Tg(-5.1myl7:DsRed-NLS) embryos at 48 hpf. Images are frontal views. Red indicates ventricle. Green indicates atrium. (C) Mean CM number from the hearts of control sibling and RARαb2 deficient Tg(-5.1myl7:DsRed-NLS) embryos at 48 hpf. (D) qPCR for CM marker gene expression in control sibling and RARαb2 deficient embryos at 48 hpf. We do find a modest decrease in CM number (C) and myl7 expression (D), which is likely due to a very modest amount of MO-induced toxicity. (TIF) [file pgen.1003689.s008.tif]

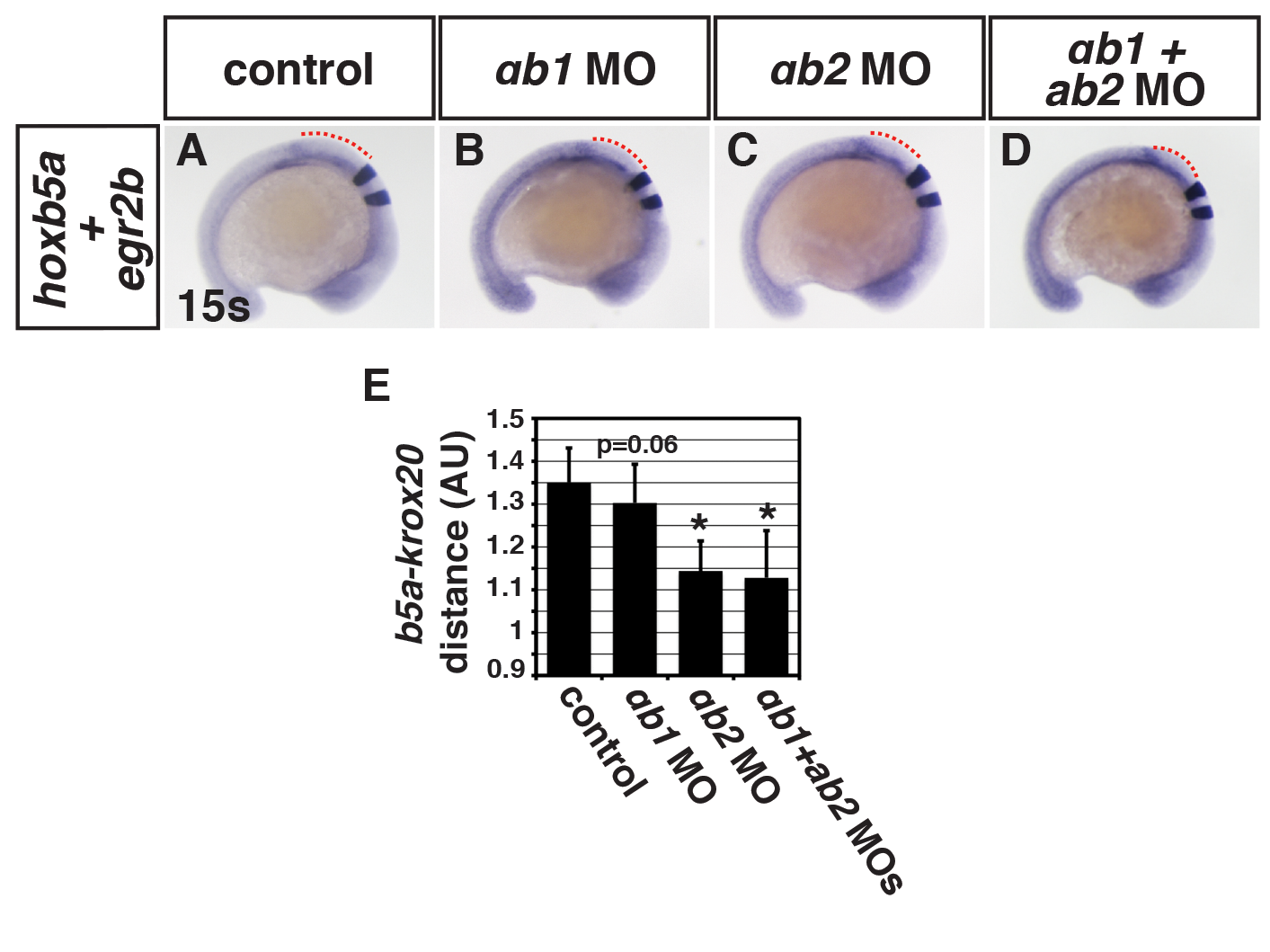

Supplement: Figure S9 — Patterning of the spinal cord is affected in the RARαb1+RARαb2 deficient embryos. (A–D) Hoxb5a (spinal cord) and egr2b (rhombomeres 3+5) expression in control (n = 32), RARαb1 deficient (n = 23), RARαb2 deficient (n = 16), and RARαb1+RARαb2 deficient embryos (n = 19). (E) Measurements of the distance in arbitrary units (AU) between hoxb5a and egr2b expression. Expression of hoxb5a in the spinal cord is expanded rostrally. The rostral expansion of hoxb5a in RARαb1 deficient embryos trends similarly as RARαb2 deficient and RARαb1+RARαb2 deficient embryos, but it is not statistically significant (p = 0.06). (TIF) [file pgen.1003689.s009.tif]

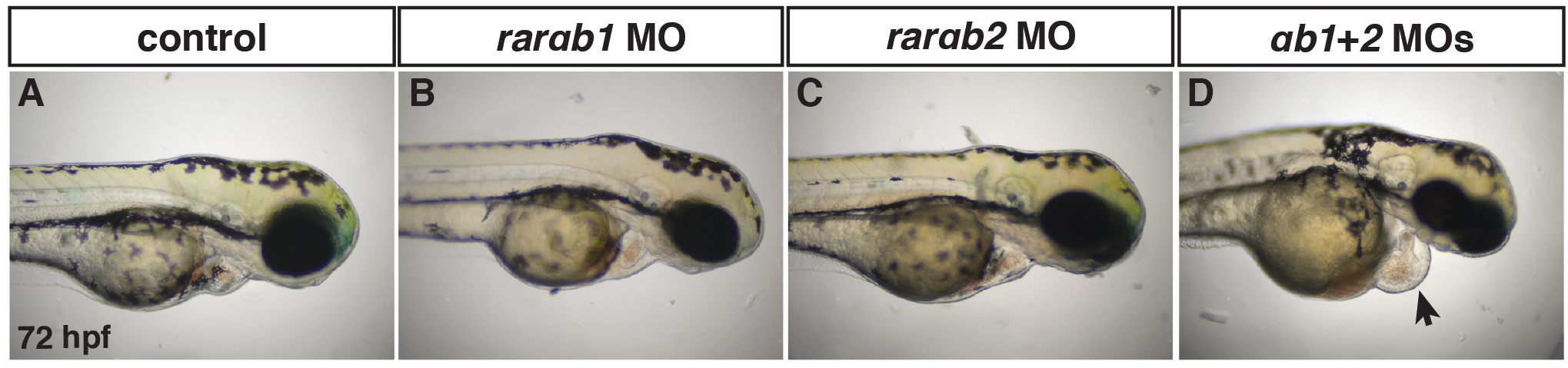

Supplement: Figure S10 — RARαb1 and RARαb2 function partially redundantly to promote proper heart development. (A–D) Control sibling, RARαb1 deficient (suboptimal dose), RARαb2 deficient (suboptimal dose), and RARαb1+RARαb2 (suboptimal doses) deficient embryos at the 72 hpf. Arrow in D indicates pericardial edema and the enlarged heart. (TIF) [file pgen.1003689.s010.tif]

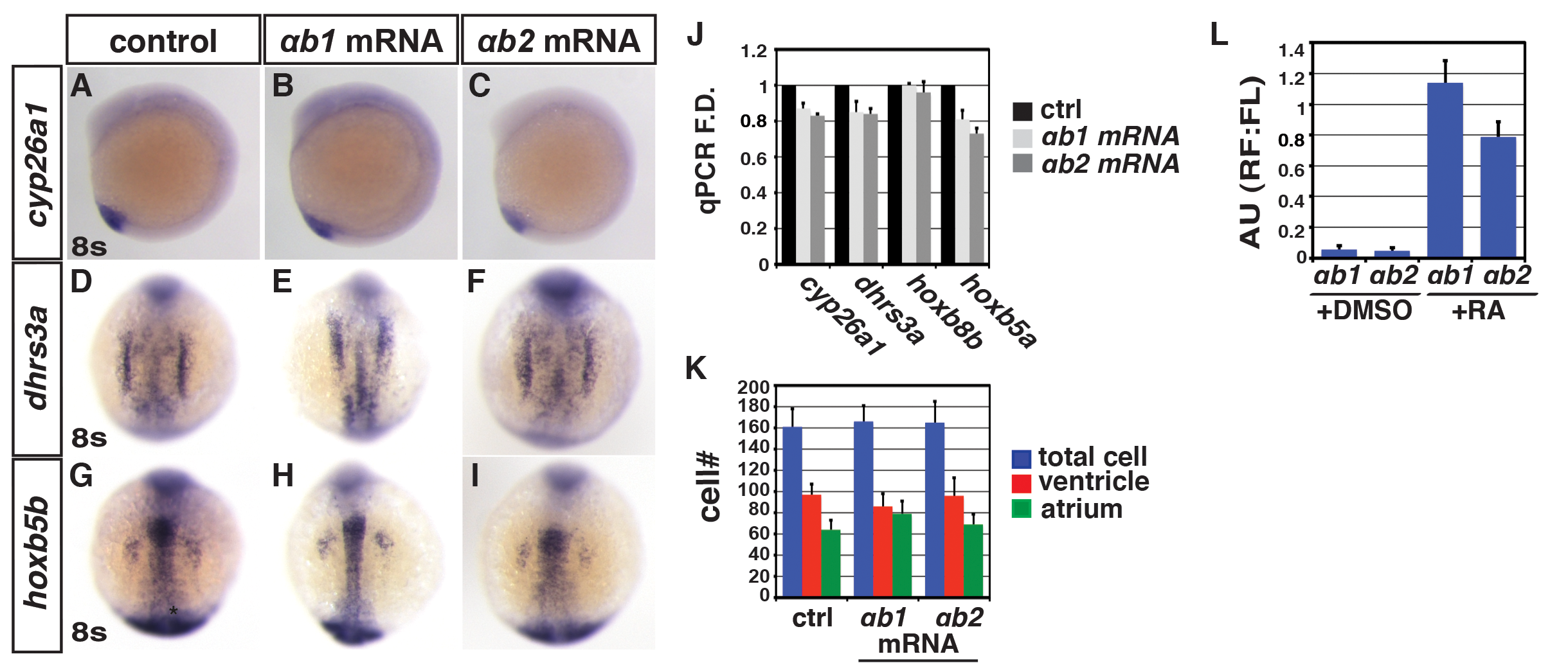

Supplement: Figure S11 — Rarαb1 and rarαb2 mRNA overexpression do not significantly affect RA responsive genes. (A–I) ISH for the RA responsive genes cyp26a1, dhrs3a, and hoxb5b at 8 s. (A, D, G) Control sibling, (B, E, H) rarαb1 mRNA, and (C, F, I) rarαb2 mRNA injected embryos. Injection of either rarαb mRNA did not inhibit RA responsive gene expression. Images in A–C are lateral views with anterior up and dorsal right. Images in D–I are dorsal views with anterior up. (J) qPCR for RA responsive genes cyp26a1, hoxb5a, hoxb8b, dhrs3a at 8 s. (K) Mean CM number from control sibling, rarαb1 mRNA, and rarαb2 mRNA injected Tg(-5.1myl7:DsRed-NLS)f2 embryos. (L) Transfection of HEK 293 cells with DNA for the zebrafish rarαb1 and rarαb2 and pGL3-12XRARE-ef1α:renilla luciferase vector with and without RA treatment. Fold difference in luminescence is indicated in arbitrary units (AU) and reflects the ratio of renilla luciferase (RL) to firefly (FL) luciferase. (TIF) [file pgen.1003689.s011.tif]

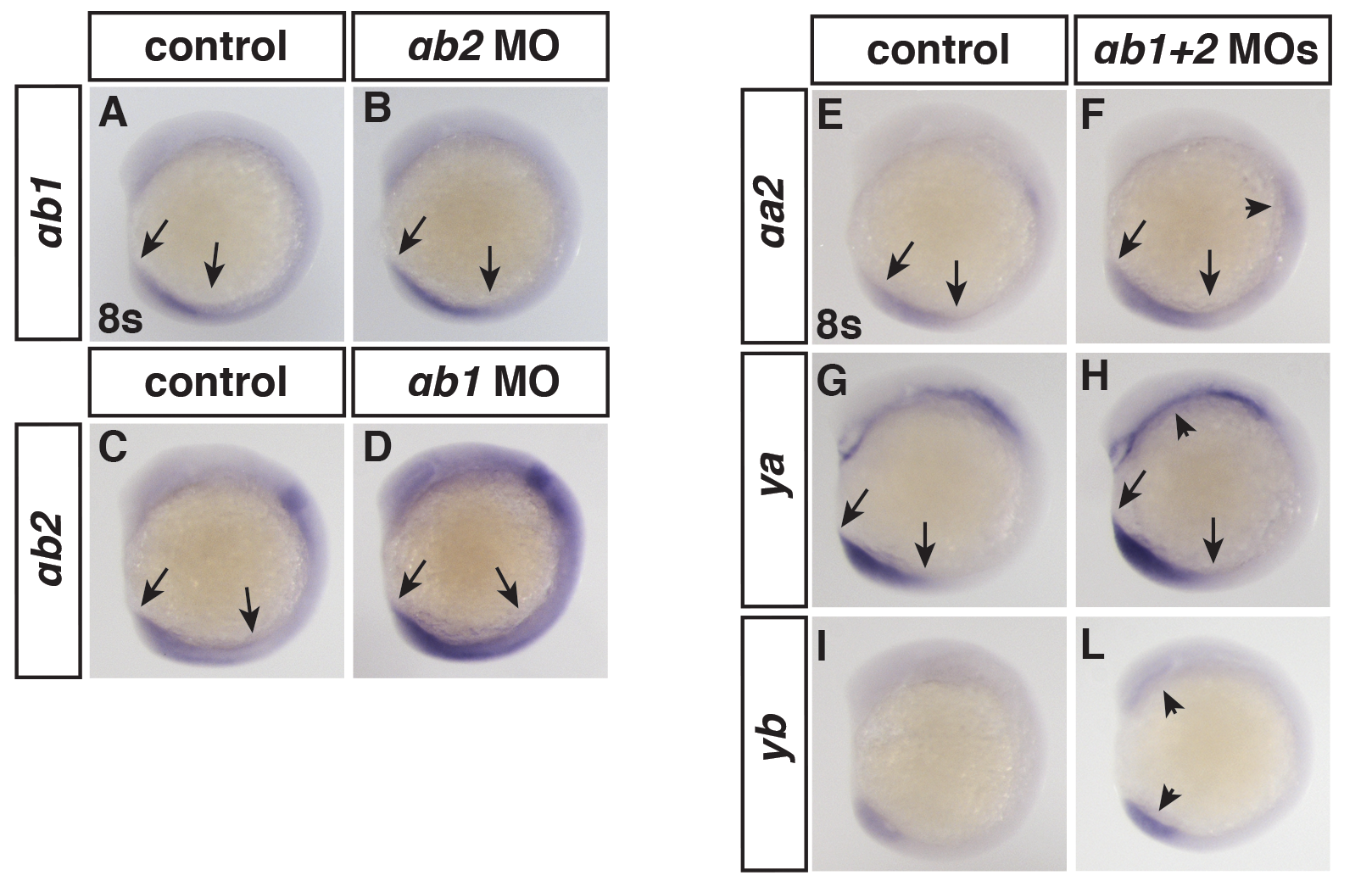

Supplement: Figure S12 — Rar expression in RARαb1, RARαb2, or RARαb1+2 deficient embryos. (A, B) ISH for rarαb1 in RARαb2 deficient embryos. (C, D) ISH for rarαb2 in RARαb1 deficient embryos. (E, F) ISH for rarαa2 in RARαb1+2 deficient embryos. (G, H) ISH for rarγa in RARαb1+2 deficient embryos. (I, L) ISH for rarγb in Rarαb1+2 deficient embryos. rar expression is often expanded in the tailbud region of embryos deficient for the other RAR homologs, while additional regions also appear to have increased or low levels of ectopic expression. All views are lateral with dorsal right at 8 s. Arrows in A–H indicate distance of expression in the tail. Arrowheads in F, H, L indicate regions of increased or ectopic expression. (TIF) [file pgen.1003689.s012.tif]

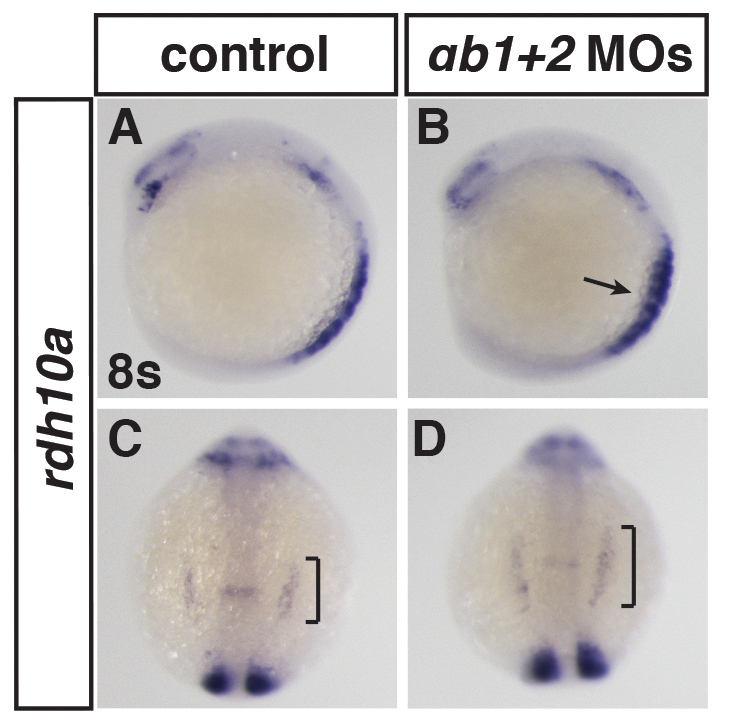

Supplement: Figure S13 — Rdh10a expression in RARαb1+2 deficient embryos. (A–D) ISH for rdh10a in RARαb1+2 deficient embryos at 8 somites. (A, B) Lateral views with dorsal right. (C, D) Dorsal views with anterior up. Brackets indicate expansion of rdh10a in the ALPM. Arrow indicates increased expression in the somites. (TIF) [file pgen.1003689.s013.tif]

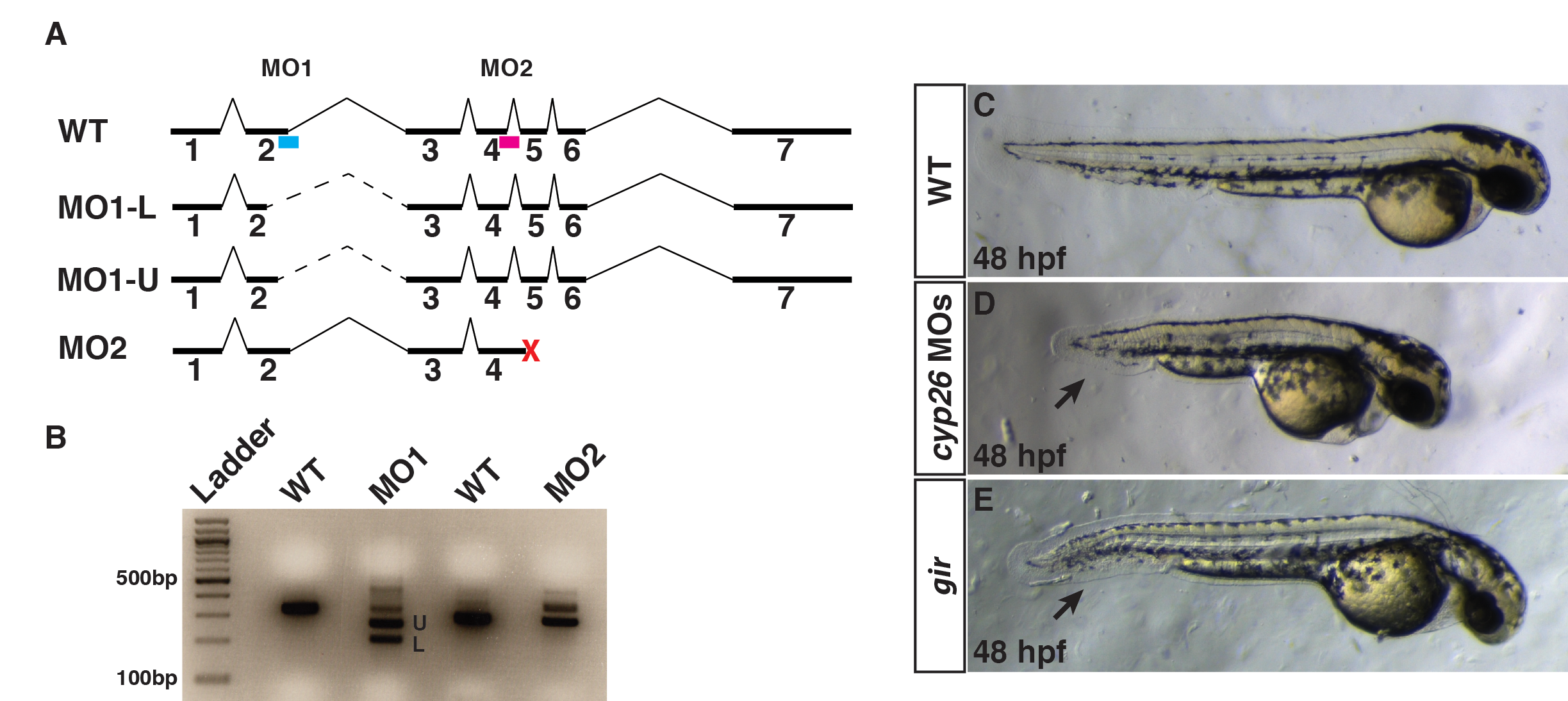

Supplement: Figure S14 — Characterization of cyp26a1 splice-blocking MOs used in experiments. (A) Schematic of the cyp26a1 locus and the intron-exon boundaries targeted by the different cyp26a1 MOs. Blue bar indicates MO1. Red bar indicates MO2. MO1 primarily causes usage of two in-frame cryptic splice sites. Dashed lines indicate the alternate introns cause by the cryptic splices induced from MO1. MO2 causes the introduction of a premature stop codon (red X). (B) RT-PCR for the WT cyp26a1 transcripts and alternate transcripts induced from the different MOs. U and L indicate bands depicted in A. (C) Control sibling embryo. (D) Embryos injected with cocktail of cyp26a1 MO1+2. Co-injection of cyp26a1 MO1 and MO2 causes a phenotype equivalent to or stronger than the cyp26a1/giraffe (gir) mutant (E). Injection of the individual MOs causes the phenotypes consistent with cyp26a1 loss of function at low frequency (data not shown). A suboptimal dose of the cyp26a1 MO cocktail was used for functional interaction experiments with RARαb1 (Figure 4). Arrows in D and E indicate shortened tail. Views in C–E are lateral with anterior right. (TIF) [file pgen.1003689.s014.tif]

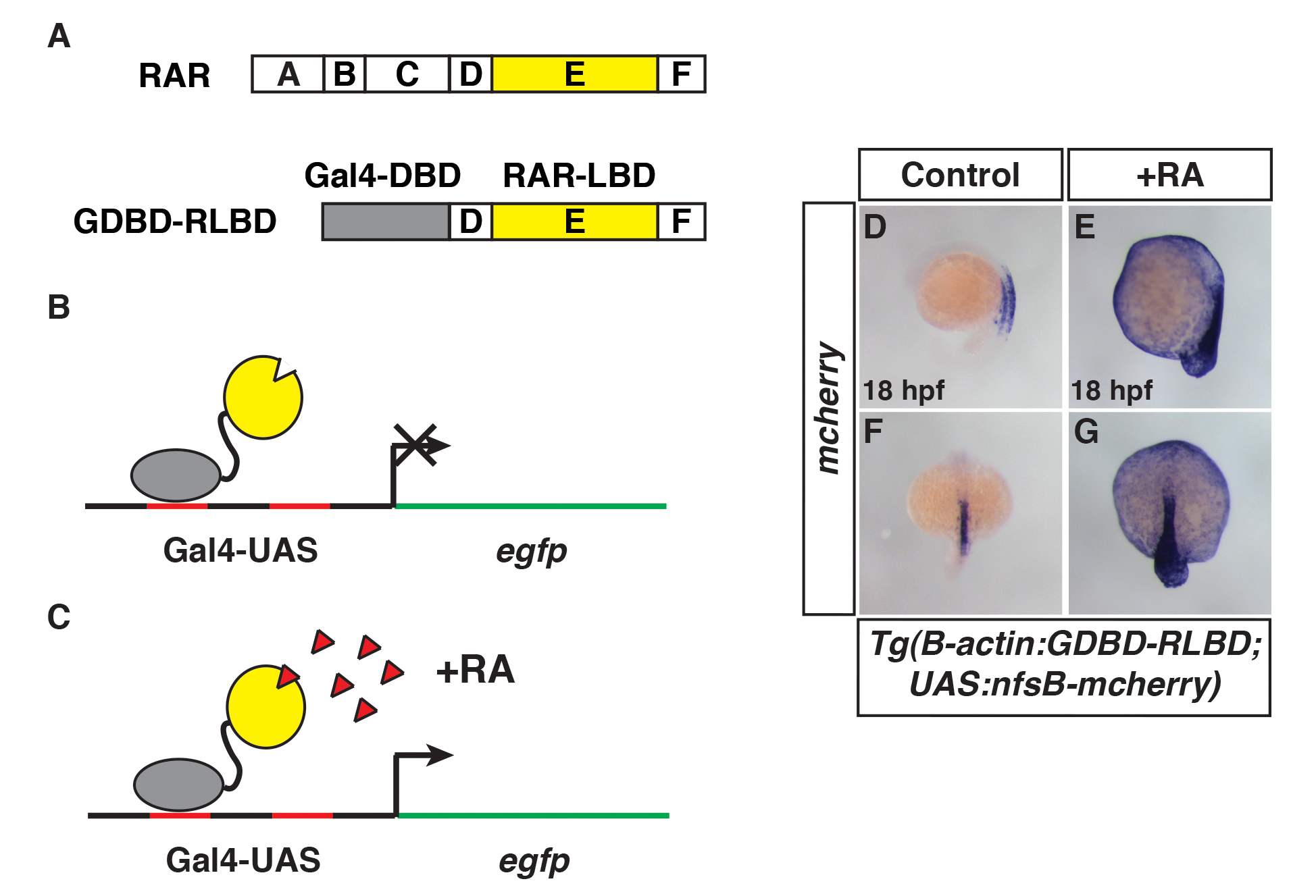

Supplement: Figure S15 — Characterization of the novel transgenic RA sensorline. (A) Schematic of the RAR domains and the Gal4 DNA binding domain (GDBD)/RARαb ligand binding domain (RLBD) fusion protein. Grey indicates the GDBD. Yellow indicates the RLBD. D is a linker domain and F is a domain with unknown function (as in Figure 1). (B, C) Schematics representing the GDBD-RLBD fusion acting on the Gal4-UAS:EGFP transgene. The GDBD-RLBD is expressed under the β-actin promoter. (B) In the absence of RA, egfp is not expressed. (C) In the presence of RA (red triangles), the GDBD-RLBD is able to promoted egfp (UAS responsive gene) transcription. (D–G) Tg(β-actin:GDBD-RLB);Tg(UAS:nfsB-mcherry) embryos are responsive to RA treatment. ISH for mcherry. Equivalent results were found when the Tg(β-actin:GDBD-RLB) line was crossed to Tg(UAS:EGFP) fish (data not shown) as were used for experiments in Figure 5. More detailed characterization of the stable transgenic RA sensor lines is reported in 31. (D, E) Lateral views with dorsal right. (F, G) Dorsal views. In images D–G anterior is up. (TIF) [file pgen.1003689.s015.tif]
